# Another Brick in the Wall of Tear Film Insights Added Through the Total Synthesis and Biophysical Profiling of *anteiso*-Branched Wax and Cholesteryl Esters

**DOI:** 10.1021/acs.jnatprod.3c01247

**Published:** 2024-03-28

**Authors:** Henrik Stubb, Tuomo Viitaja, Ryan M. Trevorah, Jan-Erik Raitanen, Jukka Moilanen, Kirsi J. Svedström, Filip S. Ekholm

**Affiliations:** † Department of Chemistry, University of Helsinki, P.O. Box 55, FI-00014 Helsinki, Finland; ‡ Ophthalmology, University of Helsinki and Helsinki University Hospital, Haartmaninkatu 8, FI-00290 Helsinki, Finland; § Department of Physics, University of Helsinki, P.O. Box 64, FI-00014 Helsinki, Finland

## Abstract

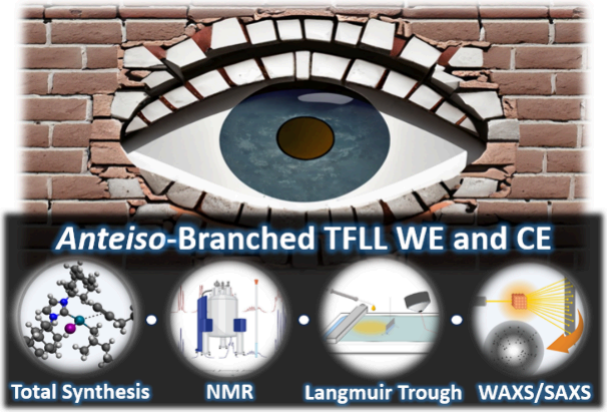

The tear film lipid layer (TFLL) plays a vital part in maintenance of ocular health and represents a unique biological barrier comprising unusual and specialized lipid classes and species. The wax and cholesteryl esters (WEs and CEs) constitute roughly 80–90% of the TFLL. The majority of species in these lipid classes are branched and it is therefore surprising that the synthesis and properties of the second largest category of species, i.e., the *anteiso*-branched species, remain poorly characterized. In this study, we have developed a total synthesis route and completed a detailed NMR spectroscopic characterization of two common *anteiso*-branched species, namely: (22*S*)-22-methyltetracosanyl oleate and cholesteryl (22′*S*)-22′-methyltetracosanoate. In addition, we have studied their structural properties in the bulk state by wide-angle and small-angle X-ray scattering and their behavior at the aqueous interface using Langmuir monolayer techniques. A comparison to the properties displayed by *iso*-branched and straight-chain analogues indicate that branching patterns lead to distinct properties in the CE and WE lipid classes. Overall, this study complements the previous work in the field and adds another important brick in the tear film insights wall.

The tear film lipid layer (TFLL), which is secreted by the meibomian glands and forms the outermost layer of the ocular surface, plays a vital role in maintenance of ocular health.^1−6^ From a biophysical perspective, the TFLL represents a unique biological barrier which is present at an interface under severe environmental stress. As a result, many of the lipid species in the TFLL have distinct molecular structures and properties compared to lipids present in other biological membranes.^7^ Supplying insights on the biophysical properties of individual tear film lipid species lays the brickwork for understanding their contributions to the molecular level organization and function of the TFLL.

Studies on the properties of human tear film lipids have in the past been hampered by limited access to such species. In more detail, the human tear film lipids are not commercially available and their isolation from human tear or meibum samples is a tedious process which does not yield individual substrates beyond minute quantities. The power of synthetic chemistry can be harnessed to circumvent these shortcomings. Recently, reports on the chemical synthesis and biophysical profiling of several key human tear film species have emerged, including *O*-acyl-ω-hydroxy fatty acids (OAHFAs), diesters, and *n*/*iso*-branched wax and cholesteryl esters (WEs and CEs).^8−10^ Nevertheless, reports on the total synthesis and biophysical profiling of tear film *anteiso*-branched WEs and CEs are absent from the literature. Insights on their properties are required to supplement the current knowledge and build an overarching understanding on the effects of chain branching on the structure and function of tear film WEs and CEs.

The WEs and CEs are the two most common types of tear film lipids and constitute roughly 80–90% of the entire TFLL (∼40–45% each).^11−14^ While the majority of studies aiming to address their properties have been performed with straight-chain species, it has been known since the early work of Andrews et.al.,^15^ and others,^16−19^ that the vast majority of WEs and CEs are in fact branched. Moreover, the proportion of branched species has been reported to deviate as a result of various ocular conditions, thus pointing toward their relevance in the function of the TFLL.^17,19−24^ In line with these observations, we recently showed that *iso*-methyl branched WEs and CEs display altered biophysical properties compared to the corresponding straight-chain ones.^9^ Herein, we set out to complete the profiling of tear film WEs and CEs by focusing on the total synthesis and biophysical profiling of selected *anteiso*-branched species.

## Results and Discussion

**Figure 1:**
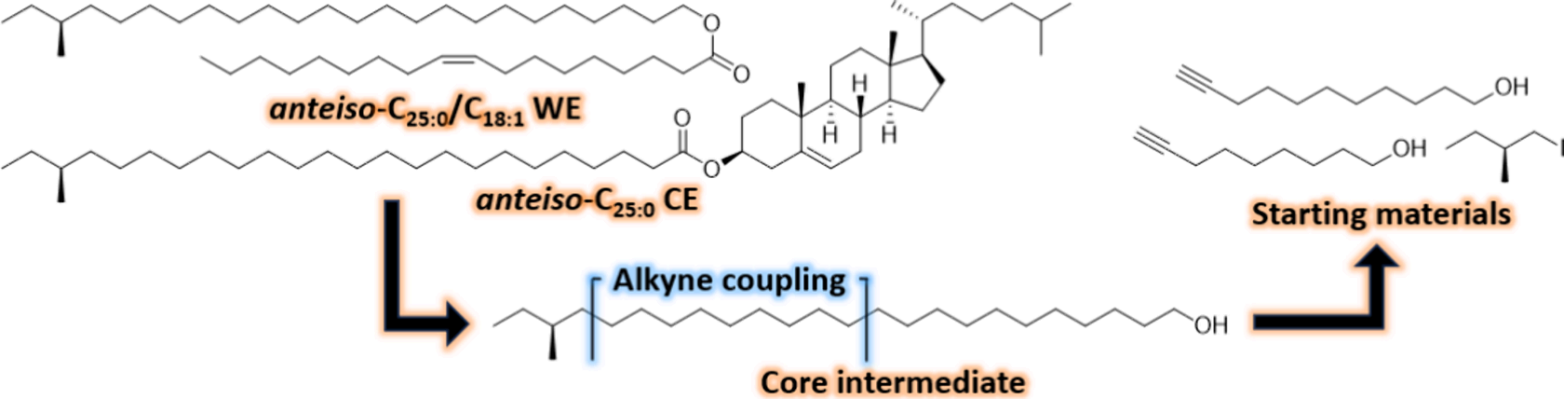
Chemical structures of the targeted *anteiso*-branched WE and CE are shown along with the retrosynthetic analysis featuring the core intermediate, starting materials and key transformations.

A great number of lipidomic profiling studies with focus on meibum and tear composition have been performed to date. We used the results from these studies as a base for selecting appropriate target molecules from the *anteiso*-branched WE and CE categories. In more detail, the *anteiso*-branched WEs and CEs constitute 23–29% of the tear film WEs and CEs.^12^ The alcohol fragment consists of predominantly saturated odd-numbered carbon chains in the range of C_21_–C_27_, whereas the most common acyl chain is oleic acid (C_18:1_).^12,16,17,24−27^ In our recent study, we focused on the total synthesis and biophysical profiling of the most prominent *iso*-branched WE (C_26:0_/C_18:1_) and CE (C_26:0_) and their corresponding straight-chain analogues. To be able to draw meaningful conclusions on the effects of distinct branching patterns on the properties of tear film lipids, target molecules with similar overall chain lengths and degrees of saturation were selected. As a result, we chose to focus on the *anteiso*-C_25:0_/C_18:1_ WE and *anteiso*-C_25:0_ CE herein. The corresponding *anteiso*-branched fatty acid and alcohol (in free form) make up roughly 2–13% of the human TFLL content,^16,17,23,26^ whereas C_25:0_/C_18:1_ WE isomer mixtures have been reported to account for 4–7%^11,28^ of the human TFLL content. While the values differ between lipidomic studies, these quantities are nevertheless high as the TFLL contains >236 individual lipid species.^11^ The molecular structures of the target molecules and a retrosynthetic analysis for their total synthesis is provided in Figure 1.

## Chemical Synthesis and Structural Characterization

### Chemical Synthesis

While limited attention has been devoted to the absolute configuration of the *anteiso*-branched species in lipidomic studies, the de novo biosynthetic pathway is known to utilize l-isoleucine as a building block which suggests that the configuration at the stereocenter is *S*.^29^ Therefore, we chose to start our synthetic route from a fragment in which the absolute configuration matched that of l-isoleucine. (*S*)-2-Methylbutanol was chosen as a suitable starting fragment. Its hydroxy group was initially converted to an iodide under modified Appel reaction conditions to obtain a suitable electrophile (**1**) for an S_N_2-displacement reaction.^30^ We originally assessed whether our previously employed alkyne coupling strategies would be suitable for the S_N_2-reaction.^9,10^ Unfortunately, the acetylide anion generated from **3** through the use of *n*-BuLi did not yield the desired product in an acceptable yield. The reason was identified as a preference for β-elimination under the employed reaction conditions. We considered that milder reaction conditions could prove more favorable and decided to investigate the potential of the Pd-catalyzed Sonogashira reaction.

The classical Sonogashira reaction is employed in the cross coupling of alkynes and aryl/allyl electrophiles under relatively mild conditions.^31^ Moreover, the bulky carbene ligand developed by Eckhardt and Fu^32^ allows similar reactions to be performed with primary alkyl halides with diminished fears concerning the formation of elimination products. At the start, we performed a brief assessment of the Sonogashira reaction with two distinct alkynyl compounds; one containing a hydroxy group (**2**) and one containing a THP-ether (**3**). A summary of the screened reaction conditions is provided in Table 1. Performing the Sonogashira reaction according to the literature protocol (Table 1, entries 1 and 2) resulted in negligible yields of the products. Isolation and characterization of the formed byproducts indicated that the low yields may have been the result of homocoupling reactions which are known to occur in this transformation.^33,34^ Unsatisfied with the yields, we decided to increase the catalyst loading to favor the Sonogashira reaction over the competing side reactions (Table 1, entries 3 and 4). With the THP-protected substrate **3**, acceptable yields in the 49% range were obtained. The yields were considerably poorer (∼16%) when the reaction was attempted with alkynol **2**. These findings indicate that the presence of different functional groups may have a considerable effect on the reaction efficiency. To explore whether the yields could be further improved, an additional attempt was made with substrate **3**, this time with an increased reaction temperature and altered solvent. Unfortunately, this resulted in a low yield of the desired product (Table 1, entry 5). Thus, our brief screening of the Sonogashira protocol enabled us to identify reaction conditions for successful coupling of **1** and **3**. The 49% yield was deemed acceptable for this work and further optimization to the reaction protocol was not performed.

**Table 1:**
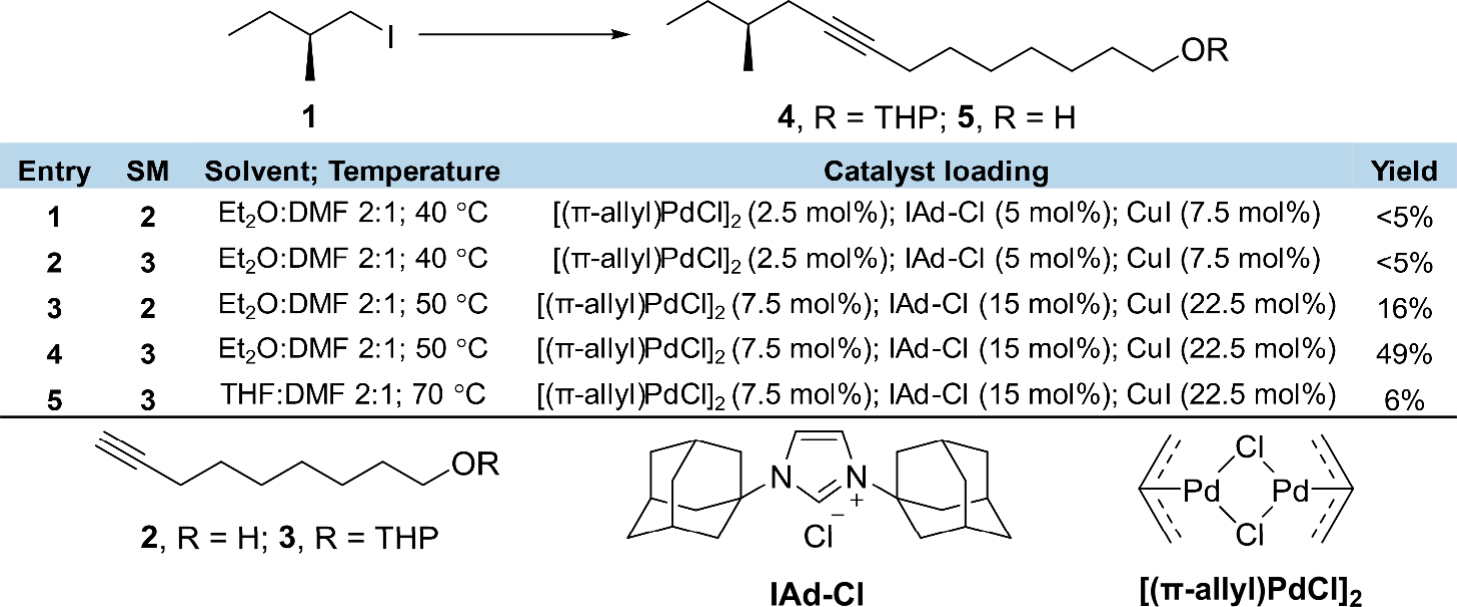
Summary of the Sonogashira Reaction Screening Performed^a^

a The reaction conditions are split between solvent/temperature and catalyst loading. SM stands for starting material and the molecular structures for key components are given below the table.

**Scheme 1:**
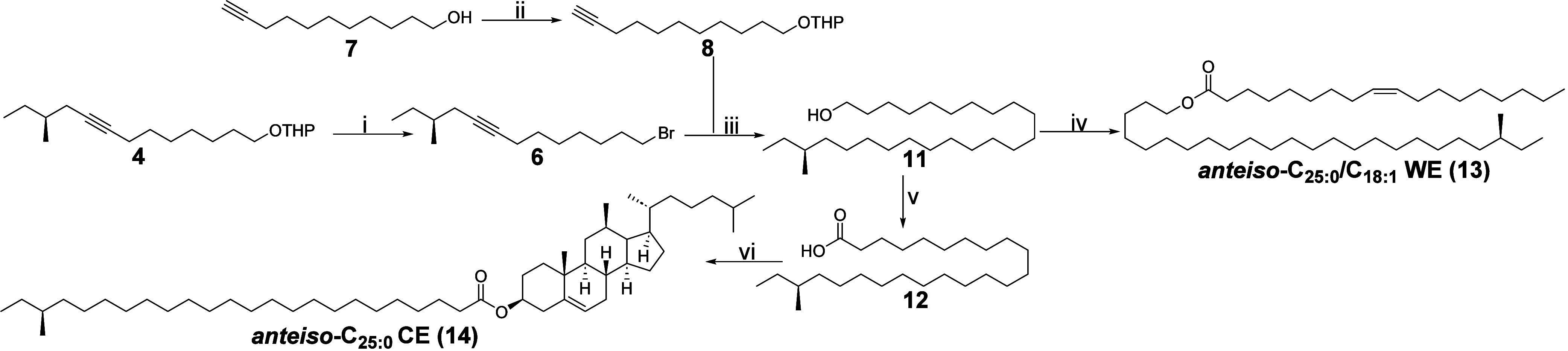
Total Synthesis of *anteiso*-C_25:0_/C_18:1_ WE (**13**) and *anteiso*-C_25:0_ CE (**14**) Reactions conditions: (i) (1) CSA, THF, MeOH, r.t., 19 h, 73%; (2) NBS, PPh_3_, CH_2_Cl_2_, r.t., 1 h, 99%; (ii) DHP, PPTS, CH_2_Cl_2_, r.t., 20 h, 97%; (iii) 1. **8**, *n*-BuLi, HMPA, THF, – 78 °C → – 40 °C, 2 h; 2. **6**, TBAI, −78 °C → 80 °C, 20 h, 85%; 3. CSA, THF, MeOH, r.t., 16 h, 81%; 4. Pd/C (10%), H_2_ (5 bar), EtOAc, r.t., 5 h, 83%; (iv) Oleic acid, EDC·HCl, DMAP, pyridine, CH_2_Cl_2_, r.t., 40 h, 87%; (v) 1. CrO_3_/H_2_SO_4_ (Jones reagent), THF, acetone, EtOAc, r.t., 1.5 h, 95%; vi) (1) SOCl_2_, DMF, toluene, 70 °C, 3 h; (2) cholesterol, DMAP, Et_3_N, r.t., 1.5 h, 62% (over two steps).

With access to **4** secured, we continued the synthetic route to *anteiso*-C_25:0_/C_18:1_ WE and *anteiso*-C_25:0_ CE. The synthetic route is summarized in Scheme 1. To reach core intermediate **11**, the length of the *anteiso*-branched carbon chain needed to be further elongated. To accomplish this, we decided to set up another alkyne coupling strategy. This time, we opted to use the more high-yielding route employed in our earlier work.^10^ In short, the THP-ether of **4** was hydrolyzed in a 73% yield under acidic conditions. The unmasked hydroxy group of **5** was converted into a bromide (**6**) in quantitative yield through an Appel-like reaction employing NBS as the bromide source. The alkyne counterpart **8** required in the coupling reaction with **6** was synthesized from commercial 10-undecyn-1-ol (**7**) through the installment of a THP-ether in 97% yield. Deprotonation of alkyne **8** with *n*-BuLi at −78 °C gave the corresponding nucleophilic acetylide anion which was subsequently reacted with the electrophilic site in **6** to yield the appropriate carbon chain length of the *anteiso*-branched core fragment. The isolated yield of the chain-elongated product **9** after workup and purification was 85%. Next, the THP ether of **9** was cleaved under acidic conditions and the triple bond of **10** was reduced employing Pd/C as the catalyst and a 5 bar H_2_-pressure. This two step protocol resulted in **11** with a 67% yield. From core intermediate **11**, the synthesis of the targeted *anteiso*-branched WE and CE was straightforward. First, the total synthesis of *anteiso*-C_25:0_/C_18:1_ WE (**13**) was successfully completed through a modified Steglich esterification reaction (DCC substituted with EDC·HCl) with oleic acid in an 87% yield.

For the total synthesis of *anteiso*-C_25:0_ CE (**14**), alcohol **11** was oxidized to the corresponding carboxylic acid **12**, i.e. (22*S*)-22-methyltetracosanoic acid, with Jones reagent. The specific rotation of this intermediate has been recorded in the excellent work by Akasaka and Ohrui^35^ which focused on the discrimination of branched fatty acids. In more detail, they reported the specific rotation for carboxylic acid **12** and provided further evidence on the enantiomeric purity through reversed-phase HPLC and labeling methods. Herein, we determined the specific rotation for carboxylic acid **12** and it matched the value provided in their report ( = +4.7 (*c* 0.48, CHCl_3_) vs  + 4.3 (*c* 0.67, CHCl_3_)) thus confirming that the stereocenter had remained intact throughout the reaction pathway. The final esterification reaction between **12** and cholesterol proved challenging. Multiple attempts employing Steglich- or Fisher-like esterification protocols resulted in poor yields due to challenging column purifications and limited reactivity. In contrast, conversion of **12** into the corresponding acyl chloride followed by direct esterification with cholesterol proved successful. Through this approach, the targeted *anteiso*-branched CE **14** could be isolated in a 62% yield.

Altogether, the total synthesis of **13** was completed in seven steps with an overall yield of 18%, whereas the total synthesis of the **14** was accomplished in eight steps with an overall yield of 12%.

### Structural Characterization

**Figure 2:**
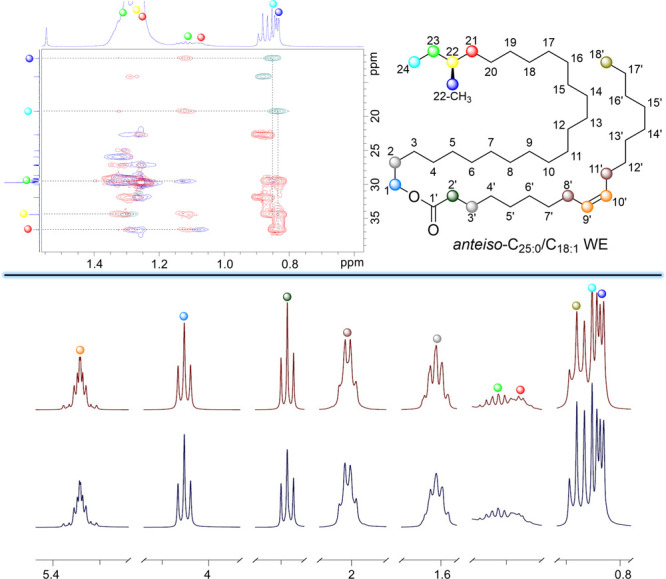
Excerpt from the NMR spectroscopic characterization work using *anteiso*-C_25:0_/C_18:1_ WE as an example. Left top panel: An overlapping Ed-HSQC and HMBC spectrum is shown together with selected signal assignments. Top right panel: The numbering used for the *anteiso*-C_25:0_/C_18:1_ WE is shown along with selected signals highlighted in the spectra (different colored balls are used). Bottom panel: the results obtained through QMSA studies are shown with selected parts of the experimental ^1^H NMR spectrum displayed on the bottom in blue and the same parts of the simulated ^1^H NMR spectrum on top in red.

The structural characterization of all reaction products (and side products when possible) was performed by MS and NMR. Special emphasis was placed on the NMR spectroscopic characterization of the products. In addition to measuring a standard set of ^1^H, ^13^C, DQF-COSY, Ed-HSQC, and HMBC spectra, quantum mechanical spectral analysis (QMSA) was performed to deduce more detailed information on chemical shifts and coupling constants. Insights on the general workflow employed in our team, and previous relevant examples, can be found in our reports on the synthesis and structural characterization of tear film lipids.^9^ The chemical shifts and coupling constants were in general found to be quite similar in the straight-chain, *iso*-branched and *anteiso*-branched WE and CE species. Therefore, we will refer the reader to our recent report on the characterization of the straight-chain and *iso*-branched species, reaction intermediates, verification of *Z*-linkages in the alkene, etc., to avoid redundant discussion herein.^9^ Instead, we will focus on the exceptions which evolve around the signals in close proximity to the branching point. Using methods such as DQF-COSY, Ed-HSQC, and HMBC, the chemical shifts of the ^1^H and ^13^C signals can be readily assigned (Figure 2, top panel). We note that the extensive work performed by Borchman and Ramasubramanian^19^ on the peak assignments in branched tear film samples are accurate and well in line with the findings of the current study. The signals in close proximity to the *anteiso*-branching site give rise to complex coupling patterns, which have not been fully solved in ultralong TFLL lipids previously. This is not surprising per se, as extracting these coupling constants is difficult without the use of dedicated QMSA-software. In this study, we used the Chemadder software for this purpose.^36^ The spin systems, chemical shifts and initial suggestive coupling constants were generated in the Chemadder interface. This starting point was optimized over multiple spectral simulation rounds until the simulated and experimental ^1^H NMR spectrum were close to identical (Figure 2, bottom panel). The chemical shifts and coupling constant for the 22-CH_3_ were similar to the values reported by Borchman and Ramasubramanian. Through the data generated with Chemadder, we propose that the coupling constants for the H-24 appearing as a dd (or t) at 0.85 ppm is 7.3–7.4 Hz to both H-23a and H-23b. These values are well in line with those reported in the literature for similar structural elements.^37−39^

Of the two neighboring diastereotopic protons H-23a and H-23b, the exact chemical shift and coupling constants could only be reliably deduced for H-23b due to the severe signal overlap in the region where H-23a is located. The coupling constants were found to be *J*_23b,23a_ = −13.7 Hz, *J*_23b,24_ = 7.3 Hz and *J*_23b,22_ = 7.8 Hz and the coupling pattern was ddq. These coupling constants are logical and in line with the ones reported for a 2-methylbutyl fragment.^40^ Similar coupling constants were also found for H-21b which appears as a dddd at 1.07 ppm (*J*_21b,21a_ = −13.9 Hz, *J*_21b,20a_ = 4.3 Hz, *J*_21b,20b_ = 6.1 Hz, *J*_21b,22_ = 8.2 Hz). With the aid of Chemadder we were able to piece together the chemical shifts and coupling constants for most of the signals near the *anteiso*-branching point. In addition, multiple other signals could be deduced as can be seen in the spectral simulation of the *anteiso*-branched WE example in Figure 2. These were the most important new findings from the NMR structural characterization point-of-view and combined with our previous reports on straight-chain and *iso*-branched species, a good foundation for future NMR-based studies in the field is now available.

### Biophysical Profiling

At the ocular surface, the TFLL forms the outermost layer of the tear film and resides on top of the aqueous tear film layer. Because the tear film is subjected to constant environmental stress, the composition needs to display adaptive features to sustain its organization and function. In order to understand the molecular level structure and interaction taking place in the TFLL, prior insights on the properties of its individual components are required. Thus, uncovering the structure and properties of distinct lipid species lays the brickwork required for a more comprehensive molecular level understanding of the TFLL. While studies on *anteiso*-branched TFLL lipids are scarce, reports on the effects of branching in general can be found. For example, studies on cell membranes of pathogens have shown that branched species can contribute to adaptive features when the membrane is subjected to environmental stress.^41−44^ In addition, our recent work on the total synthesis and biophysical profiling of *iso*-branched and straight-chain TFLL WEs and CEs showed that their melting points deviate thus affecting important biophysical properties such as film fluidity, film structure, and evaporation resistance.^9^ The results were in line with the earlier work by Rantamäki et al.^45^ and Paananen et al.^46^ who discussed extensively the connection between melting points and interfacial film behavior. Thus, we started by determining the melting points of the *anteiso*-branched WE and CE, and compared the values to those of the corresponding *iso*-branched and straight-chain analogues.

For the WEs, the melting points were: 48.0 °C for the straight-chain C_26:0_/C_18:1_ WE, 36.8 °C for the *iso*-C_26:0_/C_18:1_ WE and 27.7 °C for the *anteiso*-C_25:0_/C_18:1_ WE (**13**). Thus, branching has a notable effect on the melting points of the WEs (Table S1 of the Supporting Information, SI). In more detail, **13** has a melting point which is significantly lower than the ones of the straight-chain and *iso*-branched species. Moreover, **13** represents one of the longest *anteiso*-branched WE species in the TFLL. The fact that its melting point is substantially lower than the ocular surface temperature of 35 °C is indicative that the *anteiso*-branched WEs may be net contributors to the fluidity of the TFLL.^47^

For the CEs, the melting points were: 90.9 °C for the straight-chain C_26:0_ CE, 66.8 °C for the *iso*-C_26:0_ CE and 82.5 °C for the *anteiso*-C_25:0_ CE (**14**). While branching does lower the melting points for the CE species, the trends are distinct from those observed for the WEs (Table S1). In the case of CEs, it seems plausible that there are reinforcing interactions between the branching points and the cholesteryl moiety which affect the bulk behavior and as a result, the melting points. This is in line with some of the early studies on CEs.^48^ The fact that the melting points of the CEs are substantially higher than that of the ocular surface may indicate that they provide structural rigidity to the TFLL and are especially important in the formation of solid domains. With this initial assessment as a base, we proceeded with biophysical profiling studies focusing on the properties displayed by these lipids in the bulk state and their films at the aqueous interface. The interfacial properties were studied using a Langmuir–Blodgett trough whereas the bulk state properties were studied by wide-angle X-ray scattering (WAXS) and small-angle X-ray scattering (SAXS). It should be noted that similar techniques have previously been employed in studies on human and bovine meibum thus forming a valuable reference frame within which the properties of individual components can now be interpreted.^49,50^

#### Wide and Small Angle X-ray Scattering

The TFLL consists of several molecular layers and can from a soft matter perspective be viewed as a combination of an interface and a bulk state. Understanding the bulk state properties of tear film lipids can thus provide insights on their contributions to the crystalline domains observed in meibum.^49,51−54^ As a result, we focused our efforts on studying the crystalline nature and molecular packing in the bulk state. In this regard, WAXS and SAXS represent two valuable experimental techniques. WAXS probes the atomistic scale order, i.e., the crystalline structure and crystallinity of bulk samples whereas SAXS reveals structures at the nanometer scale such as lamellar order.

**Figure 3:**
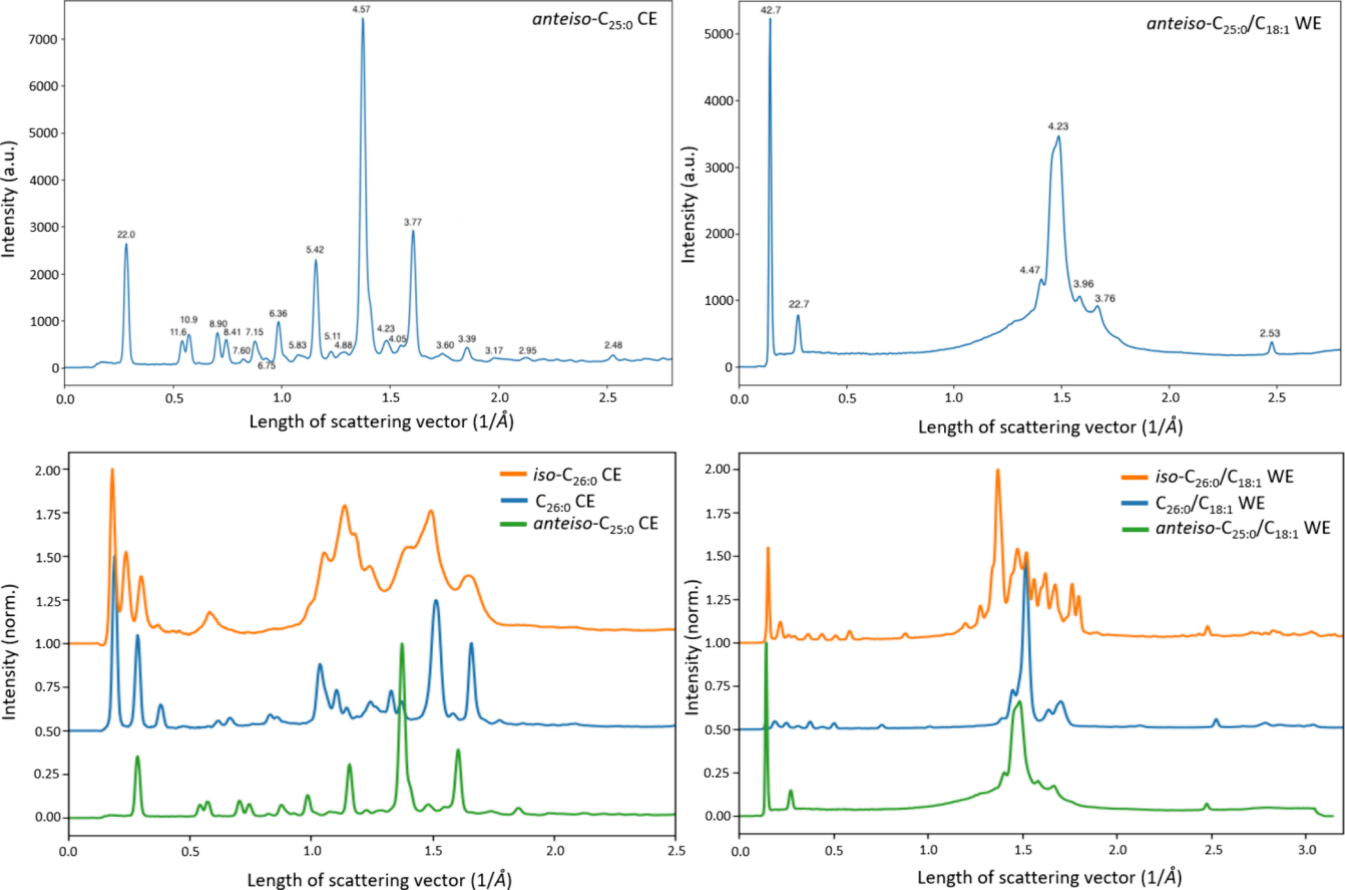
WAXS data for the straight-chain, *iso*-branched, and *anteiso*-branched WEs and CEs in bulk samples are presented. Top left, *anteiso*-C_25:0_ CE: The *d*-spacings (in units of Å) for each peak are shown, and, the long spacing was calculated to be 22.0 ± 1.0 Å. Top right, *anteiso*-C_25:0_/C_18:1_ WE: The *d*-spacings for each peak are shown, and, the long spacing was calculated to be 42.7 ± 1.0 Å. Bottom left: WAXS data for straight-chain C_26:0_ CE, *iso*-C_26:0_ CE, and *anteiso*-C_25:0_ CE demonstrate their distinct profiles. Bottom right: WAXS data for straight-chain C_26:0_/C_18:1_ WE, *iso*-C_26:0_/C_18:1_ WE, and *anteiso*-C_25:0_/C_18:1_ WE demonstrate their distinct profiles. Please note that the results from the top row are identical to the green lines which are used in the comparisons provided on the bottom row.

We started by collecting WAXS data on the bulk lipids using our laboratory setup. The processed spectra and the corresponding *d*-spacings are presented in Figure 3. Synchrotron SAXS data was also collected (Figure S25), which allowed us to confidently index the first diffraction peaks seen in the spectra and report long spacings of 42.7 ± 1.0 Å for *anteiso*-C_25:0_/C_18:1_ WE (Figure 3, top right) and 22.0 ± 1.0 Å for *anteiso*-C_25:0_ CE (Figure 3, top left). With access to WAXS data on the corresponding *iso*-branched and straight-chain species, we decided to compare our previous findings to the current ones (Figure 3, bottom left and right).

The long spacing values of *anteiso*-branched species are significantly smaller when compared to the corresponding values of *iso*-C_26:0_/C_18:1_ WE (85.3 Å) and *iso*-C_26:0_ CE (104.6 Å), and those obtained for the straight-chain C_26:0_/C_18:1_ WE (99.3 Å) and C_26:0_ CE (131.5 Å).^9^ A comparison of these values and those obtained for human meibum can be found in the SI (Table S1).^49^ In particular, the long spacings of straight-chain C_26:0_/C_18:1_ WE and *iso*-C_26:0_ CE closely resemble that of the main phase and secondary phase (49 and 111 Å, respectively) of bulk human meibum. The shorter long spacing value of *iso*-C_26:0_/C_18:1_ WE is distinct and indicative of a higher tilt angle with respect to the lamellar interface. Altogether, the previous results indicated that straight-chain C_26:0_ CE is in a bilayer crystal form, and that the *iso*-C_26:0_ CE forms an interdigitated bilayer thus resulting in a smaller long spacing value. In the *anteiso*-branched species studied herein, the long spacing of *anteiso*-C_25:0_ CE obeyed the same linear relationship as observed for the straight-chain CEs.^48^ The long spacing value of the *anteiso*-C_25:0_/C_18:1_ WE was found to be roughly that of the *iso*-C_26:0_/C_18:1_ WE (bilayer packing) divided by a factor of 2 and well in line with our previous analysis of WEs.^9^ On a general level, the long spacing values, and the bulk state packing behavior that forms the basis from which these arise, are influenced by subtle structural variations in TFLL lipid species, e.g., the site of methyl-branching, the lengths of the carbon chains and to a certain degree also chain symmetry such as the presence of odd/even numbered carbon chains.

To continue our assessment of bulk state properties, the scattering patterns observed for the different species were next compared (Figure 3). In the WE series (Figure 3, bottom right), a high degree of crystallinity (sharper peaks) was observed for the *iso*-C_26:0_/C_18:1_ WE and straight-chain C_26:0_/C_18:1_ WE species, while the *anteiso*-C_25:0_/C_18:1_ WE displayed a relatively high number of broad peaks which is indicative of a low degree of crystallinity (Figure 3, top right). In contrast, the scattering pattern of the *anteiso*-C_25:0_ CE contains a high number of particularly narrow peaks, indicative of a high degree of crystallinity (Figure 3, top left), whereas the *iso*-C_26:0_ CE displayed broad peaks and a considerably lower degree of crystallinity (Figure 3, bottom left). The straight-chain C_26:0_ CE followed a similar trend as the straight-chain C_26:0_/C_18:1_ WE and displayed a scattering pattern indicative of a high degree of crystallinity (Figure 3, bottom left).

Taken together, methyl-branching affects the melting points, crystallinity, and packing behavior of WEs and CEs in their bulk state in a distinct fashion. On the basis of the WE and CE species compared herein (most abundant species), a pool consisting of *anteiso*-branched WEs and CEs, or, *iso*-branched WEs and CEs would each supply one species with a lower degree of crystallinity and one species with a higher degree of crystallinity whereas the straight-chain WEs and CEs would only supply species with a higher degree of crystallinity. Considering that the WEs and CEs in meibum are mostly branched, it is likely that trend deviations observed in groups of *iso*/*anteiso*-WE/CE are important to maintain the adaptive features displayed by the TFLL. Nevertheless, extensive multicomponent studies with lipid libraries featuring variations in chain lengths, saturation degrees, and branching patterns will be required to delve deeper into the molecular level organization and function of the TFLL.

#### Langmuir Monolayer Studies

In the natural environment, the tear film lipids form a layer on top of the aqueous tear film layer. To understand the contributions of individual lipid components to the structure and function of the TFLL, biophysical profiling of their properties at the aqueous interface is a necessity. Herein, these studies were performed using a Langmuir–Blodgett trough. The Langmuir–Blodgett trough allows deposition of lipid species on top of the aqueous subphase thus enabling profiling of their film structure and behavior as a function of surface pressure or surface potential. In more detail, surface pressure or surface potential isotherms can be measured during compression–expansion cycles and the film structure can be imaged using a Brewster angle microscopy (BAM). Altogether, a considerable amount of information on the interfacial properties of the lipid films can be gathered under well-defined and carefully controlled conditions. In our case, we perform these studies at physiological ocular surface temperature (35 °C) utilizing an aqueous phase with similar pH and electrolyte concentration as the aqueous tear film layer.

**Figure 4:**
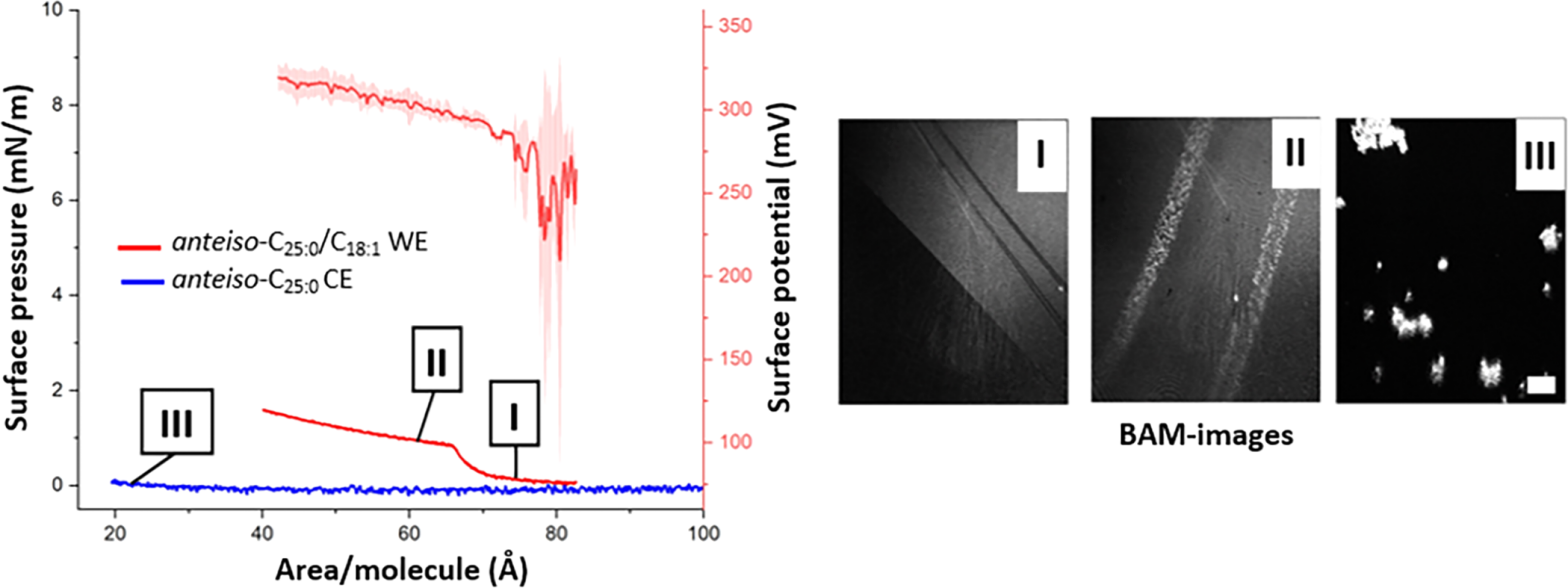
Left panel: The surface pressure and potential isotherms (±SD, *n* = 3) of *anteiso*-C_25:0_/C_18:1_ WE, and the surface pressure isotherm (±SD, *n* = 3) of *anteiso*-C_25:0_ CE as a function of area/molecule is displayed together with BAM-images at selected points on the isotherms (labels I–III). The scale bar in the BAM-images depicts 500 μm.

In our previous study on *iso*-C_26:0_/C_18:1_ and straight-chain C_26:0_/C_18:1_ CEs we noted that species with melting points above the ocular surface temperature tend to lead to formation of aggregates at the aqueous surface instead of a cohesive film under modeled physiological conditions. Therefore, it did not come as a surprise that the *anteiso*-C_25:0_ CE behaved in a similar fashion (Figure 4). In other words, *anteiso*-C_25:0_ CE was solid in its bulk state and formed aggregates at the aqueous interface at 35 °C. As a result, we consider that the most important new insights on *anteiso*-C_25:0_ CE are related to the trends uncovered in studies on bulk samples and melting points and we proceeded with studying the properties of the *anteiso*-C_25:0_/C_18:1_ WE instead.

As indicated by previous studies,^45^ the behavior of pure WE monolayers at the air–water interface is also tied to the melting points displayed by these species. For example, the straight-chain C_26:0_/C_18:1_ WE had a melting point which was substantially higher than the physiological one and formed aggregates at the aqueous interface at 35 °C whereas *iso*-C_26:0_/C_18:1_ WE had a melting point close to 35 °C and formed a multilamellar solid film which displayed a decent evaporation resistance. Nevertheless, the low melting point of the *anteiso*-C_25:0_/C_18:1_ WE indicated that this species would exist in the liquid phase at ocular surface temperature. As predicted, the BAM images proved that the *anteiso*-branched WE initially existed in the liquid phase after deposition (Figure 4, I). However, during the compression cycle we noticed that the film collapsed (bright droplets observed in the BAM images, Figure 4, II) at a low surface pressure of 0.9 mN/m which indicates that it would not be stable under the physiological surface pressure of 30 mN/m, or above.

The low collapse pressure observed was not surprising as WE films are widely known to collapse at low surface pressures. This has been attributed to challenges in balancing the polar interactions of the carbonyl group and the hydrophobic interactions caused by the lengthy hydrocarbon chains.^55^ On the whole, the surface behavior of *anteiso*-C_25:0_/C_18:1_ WE was similar to that of its shorter straight-chain analogue arachidyl oleate (AO).^56^ The film formed by the *anteiso*-C_25:0_/C_18:1_ WE did not display evaporation resistant properties on its own (data not shown) as a condensed and solid film is required to reach such features. On the basis of these findings, it seems plausible that one of the net contributions of the *anteiso*-C_25:0_/C_18:1_ WE, and other shorter *anteiso*-branched WEs, to meibum is related to lowering its melting point and increasing the fluidity of the film. These qualities might help the lipid layer in spreading and covering the aqueous tear film layer thus stabilizing the tear film. In addition, lowering of the melting point of meibum may be required in order to increase the efficiency of its secretion onto the ocular surface.

## Conclusions

The TFLL which covers the ocular surface represents a unique biological barrier which exists in a demanding environment under constant external stress and stimulus. As a result, many of the central lipid species present in the TFLL have molecular structures and properties which deviate from the lipid species present in other biological membranes. The limited access to these species has hampered studies on their properties and led to a continuous debate on the contributions of distinct lipid classes and individual components to the structural organization and adaptive function of the TFLL.

Herein, we continued on our bottom-up approach to understanding TFLL structure and function by focusing on the synthesis and biophysical profiling of *anteiso*-branched WEs and CEs thereby complementing our previous work on *iso*-branched and straight-chain analogues. In more detail, 7–8 step synthetic routes were devised and completed in 12–18% overall yields leading to the common *anteiso*-branched species; *anteiso*-C_25:0_/C_18:1_ WE (**13**) and *anteiso*-C_25:0_ CE (**14**). During the synthetic routes, all intermediates and end products were characterized in detail through NMR spectroscopic techniques coupled with QMSA and MS. For end products and key intermediates, specific rotations were also determined.

The melting points served as the first step to understanding the biophysical properties of these species in the bulk state and at the aqueous interface. In addition, WAXS and SAXS studies were performed on the bulk samples in order to uncover the degree of crystallinity in the samples and reveal the long spacing values relating to their molecular packing. The properties at the aqueous interface were studied using a Langmuir–Blodgett trough system which allows profiling of key properties as a function of surface pressure and mean molecular area at ocular surface temperature. The results from these studies were compared to our earlier findings on the *iso*-branched and straight-chain analogues of similar carbon chain lengths. Interestingly, the melting points and WAXS/SAXS data revealed distinct trends concerning the effects of *iso*- vs *anteiso*-branching on the properties of WE and CE species. The studies at the aqueous interface confirmed our previous suspicions that the *anteiso*-branched CEs may be important net contributors to rigid domains in the TFLL whereas the *anteiso*-branched WEs may contribute to its fluidity which can be important in spreading of the TFLL at the aqueous tear film layer and during the secretion of meibum onto the ocular surface.

Altogether, the preliminary biophysical profiling of the *anteiso*-branched tear film WE and CE species yielded new insights on their fundamental biophysical properties in both the bulk state and at the aqueous interface. Nevertheless, more work with tailored lipid and lipid–protein compositions will be required to reach an overarching understanding on the roles of *anteiso*-branched WEs and CEs in generation and sustenance of TFLL structure and function.

## Experimental Section

### General Experimental Procedures

Melting points were determined on a Mettler Toledo MP50 melting point device. Optical rotations were measured on a Jasco DIP-1000 digital polarimeter. NMR spectra were recorded on a Bruker Avance III instrument operating at 500 MHz (^1^H = 499.82 MHz, ^13^C = 125.68 MHz). The probe temperature was kept at 25 °C. NMR spectra were processed using the Bruker TopSpin 4.3.0 software, and the QMSA was performed with the Chemadder software (Spin Discoveries Inc.).^36^ The chemical shifts are expressed using tetramethylsilane (TMS; δ_H_ 0 ppm; δ_C_ 0 ppm) or residual solvent signals of CHCl_3_ (δ_H_ 7.26 ppm; δ_C_ 77.16 ppm) as the internal reference. Coupling constants ^*n*^*J* over distance of *n* bonds are given in Hz and provided only once when first encountered. A standard set of 1D (^1^H, ^13^C, and TOCSY) and 2D (DQF-COSY, Ed-HSQC, HMBC) NMR experiments were recorded with pulse sequences provided by the instrument manufacturer. For compounds with >250 *m*/*z*-ratio, high resolution mass spectra were recorded on either a Bruker micro Q-TOF mass spectrometer with ESI (electrospray ionization), ThermoScientific Q Exactive HF Orbitrap MS with heated-electrospray ionization (H-ESI), or Shimadzu AXIMA Performance mass spectrometer with MALDI-TOF (matrix-assisted laser-desorption-ionization time-of-flight), all operated in positive mode. Compounds with lower *m*/*z*-ratio were analyzed by GC–MS on a Bruker Scion 456-GC (column: DB-5ms, 5% phenylmethylsiloxane, 30 m × 0.25 mm i.d.; carrier gas: He, 1.3 mL/min; pressure: 97.9 kPa; Temperature program A: 135 °C (4 min), then 5 °C/min to 280 °C (10 min); Temperature program B: 40 °C (1 min), then 6 °C/min to 230 °C (5 min)) with single quadrupole mass analyzer and electron ionization (EI, 70 eV). TLC was performed on aluminum sheets precoated with silica gel 60 F254 (Merck). Unless otherwise mentioned, spots were visualized by spraying the plates with a 5:1 MeOH/H_2_SO_4_-solution followed by charring. Flash chromatography was carried out on silica gel 40 (40–63 μm, Merck). All chemicals were purchased from commercial sources and were used as obtained without further purification. Reactions involving moisture and/or air sensitive reagents were carried out under an argon atmosphere. Dry VAC purified solvents and standard Schlenk techniques were used when appropriate.

### General Procedure for Sonogashira Coupling of Alkyl Halides

IAd-Cl (5.0 mol %), CuI (7.5 mol %), [(π-allyl)PdCl]_2_ (2.5 mol %) and Cs_2_CO_3_ (1.4 equiv) were heated inside an airtight flask which was evacuated and flushed with argon. Using standard Schlenk techniques, a mixture of dry and degassed DMF:Et_2_O (2:1, 3 mL/1.5 mmol of halide) was added. To the yellow mixture was added the terminal alkyne (1.3 equiv) and alkyl halide (1.0 equiv), resulting in a black mixture. The heterogeneous mixture was heated for 21 h and then quenched with a saturated aqueous NH_4_Cl-solution (20 mL/1.5 mmol of halide). The aqueous phase was extracted with Et_2_O (5 × 20 mL). The organic phases were combined, dried over anhydrous Na_2_SO_4_, and filtered through Diatomaceous earth. The Diatomaceous earth was washed with Et_2_O (100 mL/1.5 mmol of halide) and the filtrate was evaporated under reduced pressure. The product was purified by silica gel flash chromatography (*n*-hexane:EtOAc 98:2 → 9:1) and dried in vacuo.

#### (*S*)-1-Iodo-2-methylbutane (1)

(*S*)-2-Methylbutan-1-ol (1.01 g, 11.4 mmol, 1.0 equiv) was dissolved in dry CH_2_Cl_2_ (16 mL), and Ph_3_P (3.59 g, 13.7 mmol, 1.2 equiv) and NIS (3.08 g, 13.7 mmol, 1.2 equiv) were added in small portions at 0 °C. The mixture was stirred at r.t. under argon for 4 h and then filtered through a pad of silica. The silica was rinsed with *n*-pentane (150 mL), and the filtrate was concentrated under reduced pressure. The product was purified by silica gel flash chromatography (pentane) and dried under reduced pressure (700 mbar, 40 °C) to obtain a clear, colorless volatile liquid (1.86 g, 82%).  + 4.4 (*c* 2.00, *n*-pentane). *R*_f_ = 0.97 (*n*-pentane, visualized by UV-lamp λ = 254 nm).

^1^H NMR (499.82 MHz, CDCl_3_, 25 °C): δ_H_ 3.23 (1H, dd, ^2^*J*_1a,1b_ = −9.6 Hz, ^3^*J*_1a,2_ = 4.7 Hz, H-1a), 3.17 (1H, dd, ^3^*J*_1b,2_ = 6.0 Hz, H-1b), 1.42 (1H, ddq, ^2^*J*_3a,3b_ = −13.3 Hz, ^3^*J*_3a,2_ = 6.1 Hz, ^3^*J*_3a,4_ = 7.7 Hz, H-3a), 1.38 (1H, ddddq, ^3^*J*_2,3b_ = 6.8 Hz, ^3^*J*_2,2-CH_3__ = 6.6 Hz, H-2), 1.26 (1H, ddq, ^3^*J*_3b,4_ = 7.5 Hz, H-3b), 0.98 (3H, d, 2-CH_3_) and 0.89 (3H, t, H-4) ppm.

^13^C NMR (125.68 MHz, CDCl_3_, 25 °C): δ_C_ 36.41 (CH, C-2), 29.21 (CH_2_, C-3), 20.18 (CH_3_, 2-CH_3_), 17.45 (CH_2_, C-1), and 11.30 (CH_3_, C-4) ppm.

GC-MS temperature program B *t*_R_ = 6.82 min: *m*/*z* 197.9 (1) [M^+^, C_5_H_11_I], 168.8 (3), 140.7 (3), 126.8 (14), 71.1 (100).

#### 1-(2′-Tetrahydropyranyloxy)-8-nonyne (3)

8-Nonyn-1-ol (**2**, 4.65 g, 33.2 mmol, 1.0 equiv) was dissolved in dry CH_2_Cl_2_ (65 mL), and DHP (5.57 g, 66.2 mmol, 2.0 equiv) and PPTS (835 mg, 3.3 mmol, 0.1 equiv) were added. The mixture was stirred at r.t. under argon for 20 h and quenched with H_2_O (60 mL). The aqueous phase was extracted with CH_2_Cl_2_ (2 × 60 mL). The organic phases were combined, dried over anhydrous Na_2_SO_4_, filtered, and evaporated under reduced pressure. The product was purified by silica gel flash chromatography (*n*-hexane:EtOAc 95:5 → 9:1) and dried in vacuo to obtain a clear, colorless liquid (7.28 g, 98%). *R*_f_ = 0.44 (*n*-hexane:EtOAc 9:1).

^1^H NMR (499.82 MHz, CDCl_3_, 25 °C): δ_H_ 4.57 (1H, dd, ^3^*J*_2′,3′a_ = 2.7 Hz, ^3^*J*_2′,3′b_ = 4.5 Hz, H-2′), 3.87 (1H, ddd, ^2^*J*_6′a,6′b_ = −11.2 Hz, ^3^*J*_6′a,5′b_ = 2.9 Hz, ^3^*J*_6′a,5′a_ = 8.2 Hz, H-6′a), 3.73 (1H, dt, ^3^*J*_1a,2_ = 6.9 Hz, ^2^*J*_1a,1b_ = 9.6 Hz, H-1a), 3.50 (1H, ddd, ^3^*J*_6′b,5′b_ = 3.6 Hz, ^3^*J*_6′b,5′a_ = 7.0 Hz, H-6′b), 3.38 (1H, dt, ^3^*J*_1b,2_ = 6.6 Hz, H-1b), 2.18 (2H, dt, ^4^*J*_7,9_ = −2.6 Hz, ^3^*J*_7,6_ = 7.1 Hz, H-7), 1.93 (1H, t, H-9), 1.83 (1H, ddddd, ^2^*J*_4′a,4′b_ = −11.8 Hz, ^3^*J*_4′a,5′b_ = 3.1 Hz, ^3^*J*_4′a,3′b_ = 3.2 Hz, ^3^*J*_4′a,5′a_ = 7.9 Hz, ^3^*J*_4′a,3′a_ = 9.3 Hz, H-4′a), 1.71 (1H, dddd, ^2^*J*_3′a,3′b_ = −12.7 Hz, ^3^*J*_3′a,4′b_ = 4.4 Hz, H-3′a), 1.60 (2H, ddt, ^3^*J*_2,3_ = 7.7 Hz, H-2), 1.59–1.49 (6H, m, H-3′b, H-4′b, H-5′a, H-5′b, H-6) and 1.44–1.27 (6H, m, H-3–H-5) ppm; ^13^C NMR (125.68 MHz, CDCl_3_, 25 °C): δ_C_ 98.9 (CH, C-2′), 84.7 (C, C-8), 68.1 (CH, C-9), 67.6 (CH_2_, C-1), 62.3 (CH_2_, C-6′), 30.8 (CH_2_, C-3′), 29.7 (CH_2_, C-2), 28.9 (CH_2_, C-4), 28.7 (CH_2_, C-5), 28.4 (CH_2_, C-6), 26.1 (CH_2_, C-3), 25.5 (CH_2_, C-5′), 19.7 (CH_2_, C-4′), and 18.4 (CH_2_, C-7) ppm; GC-MS temperature program A *t*_R_ = 11.50 min: *m*/*z* 224.3 (0.1) [M^+^, C_14_H_24_O_2_], 223.2 (0.2), 151.0 (0.1), 135.1 (0.2), 123.0 (0.7), 111.0 (1), 107.0 (2), 101.0 (20), 93.0 (4), 85.0 (100).

#### (11*S*)-1-(2′-Tetrahydropyranyloxy)-11-methyltridec-8-yne (4)

The product was synthesized according to the general procedure for Sonogashira coupling of alkyl halides from **3** (418 mg, 1.86 mmol, 1.3 equiv) and **1** (289 mg, 1.46 mmol, 1.0 equiv). Catalyst loadings were tripled. Title compound was obtained as a clear, colorless oil (211 mg, 49%). *R*_f_ = 0.47 (*n*-hexane:EtOAc 9:1).

^1^H NMR (499.82 MHz, CDCl_3_, 25 °C): δ_H_ 4.57 (1H, dd, ^3^*J*_2′,3′a_ = 2.8 Hz, ^3^*J*_2′,3′b_ = 4.6 Hz, H-2′), 3.87 (1H, ddd, ^2^*J*_6′a,6′b_ = −11.1 Hz, ^3^*J*_6′a,5′b_ = 2.9 Hz, ^3^*J*_6′a,5′a_ = 8.2 Hz, H-6′a), 3.73 (1H, dt, ^2^*J*_1a,1b_ = −9.6 Hz, ^3^*J*_1a,2_ = 6.9 Hz, H-1a), 3.50 (1H, ddd, ^3^*J*_6′b,5′b_ = 3.6 Hz, ^3^*J*_6′b,5′a_ = 7.0 Hz, H-6′b), 3.38 (1H, dt, ^3^*J*_1b,2_ = 6.7 Hz, H-1b), 2.15 (2H, ddt, ^5^*J*_7,10a_ = 2.3 Hz, ^5^*J*_7,10b_ = 2.3 Hz, ^3^*J*_7,6_ = 6.9 Hz, H-7), 2.12 (1H, ddt, ^2^*J*_10a,10b_ = −16.3 Hz, ^3^*J*_10a,11_ = 5.7 Hz, H-10a), 2.03 (1H, ddt, ^3^*J*_10b,11_ = 6.9 Hz, H-10b), 1.83 (1H, ddddd, ^2^*J*_4′a,4′b_ = −11.9 Hz, ^3^*J*_4′a,5′b_ = 3.1 Hz, ^3^*J*_4′a,3′b_ = 3.6 Hz, ^3^*J*_4′a,5′a_ = 7.9 Hz, ^3^*J*_4′a,3′a_ = 9.3 Hz, H-4′a), 1.71 (1H, dddd, ^2^*J*_3′a,3′b_ = −12.7 Hz, ^3^*J*_3′a,4′b_ = 4.3 Hz, H-3′a), 1.60 (2H, ddt, ^3^*J*_2,3_ = 7.9 Hz, H-2), 1.59–1.29 (14H, m, H-3′b, H-4′b, H-5′a, H-5′b, H-3–H-6, H-11, H-12a), 1.21 (1H, ddq, ^2^*J*_12b,12a_ = −13.8 Hz, ^3^*J*_12b,13_ = 7.3 Hz, ^3^*J*_12b,11_ = 7.4 Hz, H-12b), 0.95 (3H, d, ^3^*J*_11-CH3,11_ = 6.7 Hz, 11-CH_3_), and 0.88 (3H, dd, ^3^*J*_13,12a_ = 7.6 Hz, H-13) ppm.

^13^C NMR (125.68 MHz, CDCl_3_, 25 °C): δ_C_ 98.9 (CH, C-2′), 81.0 (C, C-8), 79.0 (C, C-9), 67.6 (CH_2_, C-1), 62.3 (CH_2_, C-6′), 34.6 (CH, C-11), 30.8 (CH_2_, C-3′), 29.7 (CH_2_, C-2), 29.1 (CH_2_, C-6), 29.0 (CH_2_, C-4), 28.8 (CH_2_, C-5), 28.7 (CH_2_, C-12), 26.2 (CH_2_, C-3), 25.8 (CH_2_, C-10), 25.5 (CH_2_, C-5′), 19.7 (CH_2_, C-4′), 19.1 (CH_3_, 11-CH_3_), 18.8 (CH_2_, C-7), and 11.5 (CH_3_, C-13) ppm; HRESIMS *m*/*z* 317.2584 [M + Na]^+^ (calcd for C_19_H_34_O_2_Na, 317.2451).

#### (11*S*)-11-Methyltridec-8-yn-1-ol (5)

**4** (380 mg, 1.29 mmol, 1.0 equiv) was dissolved in dry THF (6 mL) and dry MeOH (12 mL), and CSA (32 mg, 0.14 mmol, 0.1 equiv) was added. The mixture was stirred at r.t. under argon for 20 h and then concentrated under reduced pressure. The product was purified by silica gel flash chromatography (*n*-hexane:EtOAc 9:1 → 4:1) and dried in vacuo to obtain a clear, colorless liquid (199 mg, 73%). *R*_f_ = 0.39 (*n*-hexane:EtOAc 4:1).

^1^H NMR (499.82 MHz, CDCl_3_, 25 °C): δ_H_ 3.64 (2H, dt, ^3^*J*_1,OH_ = 3.2 Hz, ^3^*J*_1,2_ = 6.4 Hz, H-1), 2.15 (2H, ddt, ^5^*J*_7,10b_ = 2.3 Hz, ^5^*J*_7,10a_ = 2.4 Hz, ^3^*J*_7,6_ = 6.8 Hz, H-7), 2.12 (1H, ddt, ^2^*J*_10a,10b_ = −16.4 Hz, ^3^*J*_10a,11_ = 5.7 Hz, H-10a), 2.03 (1H, ddt, ^3^*J*_10b,11_ = 6.9 Hz, H-10b), 1.57 (2H, tt, ^3^*J*_2,3_ = 7.9 Hz, H-2), 1.56–1.29 (11H, m, H-3–H-6, H-11, H12-a), 1.27 (1H, d, 1-OH), 1.21 (1H, ddq, ^2^*J*_12b,12a_ = −13.4 Hz, ^3^*J*_12b,13_ = 7.3 Hz, ^3^*J*_12b,11_ = 7.5 Hz, H-12b), 0.95 (3H, d, ^3^*J*_11-CH3,11_ = 6.6 Hz, 11-CH_3_), and 0.88 (3H, dd, ^3^*J*_13,12a_ = 7.5 Hz, H-13) ppm.

^13^C NMR (125.68 MHz, CDCl_3_, 25 °C): δ_C_ 80.9 (C, C-8), 79.0 (C, C-9), 63.0 (CH_2_, C-1), 34.6 (CH, C-11), 32.8 (CH_2_, C-2), 29.1 (CH_2_, C-6), 28.9 (CH_2_, C-4), 28.8 (CH_2_, C-5), 28.7 (CH_2_, C-12), 25.8 (CH_2_, C-10), 25.7 (CH_2_, C-3), 19.1 (CH_3_, 11-CH_3_), 18.7 (CH_2_, C-7), and 11.5 (CH_3_, C-13) ppm.

GC-MS temperature program A *t*_R_ = 12.30 min: *m*/*z* 210.2 (1) [M^+^, C_14_H_26_O], 195.2 (2), 181.2 (4), 178.9 (5), 163.1 (16), 153.1 (10), 139.3 (11), 135.1 (100).

#### (11*S*)-1-Bromo-11-methyltridec-8-yne (6)

**5** (209 mg, 0.99 mmol, 1.0 equiv) was dissolved in dry CH_2_Cl_2_ (16 mL), and Ph_3_P (312 mg, 1.19 mmol, 1.2 equiv) and NBS (212 mg, 1.19 mmol, 1.2 equiv) were added at 0 °C. The mixture was stirred at r.t. under argon for 1.5 h, then diluted with *n*-hexane:EtOAc 9:1 (50 mL), and filtered through a pad of silica. The silica was rinsed with *n*-hexane:EtOAc 9:1 (150 mL), and the filtrate was concentrated under reduced pressure. The product was purified by silica gel flash chromatography (*n*-hexane:EtOAc 9:1) and dried in vacuo to obtain a clear, colorless liquid (271 mg, > 99%). *R*_f_ = 0.83 (*n*-hexane:EtOAc 9:1).

^1^H NMR (499.82 MHz, CDCl_3_, 25 °C): δ_H_ 3.41 (2H, t, ^3^*J*_1,2_ = 6.9 Hz, H-1), 2.15 (2H, ddt, ^5^*J*_7,10a_ = 2.4 Hz, ^5^*J*_7,10b_ = 2.5 Hz, ^3^*J*_7,6_ = 7.0 Hz, H-7), 2.12 (1H, ddt, ^2^*J*_10a,10b_ = −16.4 Hz, ^3^*J*_10a,11_ = 5.7 Hz, H-10a), 2.03 (1H, ddt, ^3^*J*_10b,11_ = 6.9 Hz, H-10b), 1.86 (2H, tt, ^3^*J*_2,3_ = 7.7 Hz, H-2), 1.52 (1H, ddddq, ^3^*J*_11,12a_ = 5.2 Hz, ^3^*J*_11,11-CH3_ = 6.6 Hz, ^3^*J*_11,12b_ = 7.5 Hz, H-11), 1.49 (2H, tt, H-6), 1.48–1.37 (5H, m, H-3, H-5, H-12a), 1.33 (2H, tt, ^3^*J*_4,3_ = 7.4 Hz, ^3^*J*_4,5_ = 7.8 Hz, H-4), 1.21 (1H, ddq, ^2^*J*_12b,12a_ = −13.5 Hz, ^3^*J*_12b,13_ = 7.3 Hz, H-12b), 0.95 (3H, d, 11-CH_3_), and 0.88 (3H, dd, ^3^*J*_13,12a_ = 7.5 Hz, H-13) ppm.

^13^C NMR (125.68 MHz, CDCl_3_, 25 °C): δ_C_ 80.8 (C, C-8), 79.1 (C, C-9), 34.6 (CH, C-11), 33.9 (CH_2_, C-1), 32.8 (CH_2_, C-2), 29.0 (CH_2_, C-6), 28.7 (CH_2_, C-12), 28.6 (CH_2_, C-5), 28.3 (CH_2_, C-4), 28.1 (CH_2_, C-3), 25.8 (CH_2_, C-10), 19.1 (CH_3_, 11-CH_3_), 18.7 (CH_2_, C-7) and 11.5 (CH_3_, C-13) ppm.

GC-MS temperature program *t*_R_ = 14.18 min: *m*/*z* 272.1 (0.3) [M^+^, C_14_H_25_Br], 257.1 (0.1), 243.1 (2), 215.1 (4), 201.1 (5), 189.0 (5), 172.9 (4), 163.2 (3), 135.0 (15), 121.0 (14), 109.0 (100).

#### 1-(2′-Tetrahydropyranyloxy)-10-undecyne (8)

10-Undecyn-1-ol (**7**, 3.64 g, 21.2 mmol, 1.0 equiv) was dissolved in dry CH_2_Cl_2_ (50 mL), and DHP (3.65 g, 43.3 mmol, 2.0 equiv) and PPTS (547 mg, 2.2 mmol, 0.1 equiv) were added. The mixture was stirred at r.t. under argon for 21 h and quenched with H_2_O (50 mL). The aqueous phase was extracted with CH_2_Cl_2_ (2 × 50 mL). The organic phases were combined, dried over anhydrous Na_2_SO_4_, filtered, and evaporated under reduced pressure. The product was purified by silica gel flash chromatography (*n*-hexane:EtOAc 95:5) and dried *in vacuo* to obtain a clear, colorless liquid (5.18 g, 97%). *R*_*f*_ = 0.46 (*n*-hexane:EtOAc 9:1).

^1^H NMR (499.82 MHz, CDCl_3_, 25 °C): δ_H_ 4.57 (1H, dd, ^3^*J*_2′,3′a_ = 2.7 Hz, ^3^*J*_2′,3′b_ = 4.5 Hz, H-2’), 3.87 (1H, ddd, ^2^*J*_6’a,6’b_ = −11.2 Hz, ^3^*J*_6’a,5′b_ = 2.6 Hz, ^3^*J*_6’a,5′a_ = 8.2 Hz, H-6’a), 3.73 (1H, dt, ^2^*J*_1a,1b_ = −9.6 Hz, ^3^*J*_1a,2_ = 7.0 Hz, H-1a), 3.50 (1H, ddd, ^3^*J*_6’b,5′b_ = 3.6 Hz, ^3^*J*_6’b,5′a_ = 7.0 Hz, H-6’b), 3.38 (1H, dt, ^3^*J*_1b,2_ = 6.7 Hz, H-1b), 2.18 (2H, dt, ^4^*J*_9,11_ = −2.6 Hz, ^3^*J*_9,8_ = 7.1 Hz, H-9), 1.94 (1H, t, H-11), 1.83 (1H, ddddd, ^2^*J*_4’a,4’b_ = −11.8 Hz, ^3^*J*_4’a,5′b_ = 3.1 Hz, ^3^*J*_4’a,3′b_ = 3.2 Hz, ^3^*J*_4’a,5′a_ = 7.9 Hz, ^3^*J*_4’a,3′a_ = 9.3 Hz, H-4’a), 1.71 (1H, dddd, ^2^*J*_3′a,3′b_ = −12.8 Hz, ^3^*J*_3′a,4’b_ = 3.2 Hz, H-3′a), 1.59 (2H, ddt, ^3^*J*_2,3_ = 7.7 Hz, H-2), 1.59–1.48 (6H, m, H-3′b, H-4’b, H-5′a, H-5′b, H-8) and 1.42–1.27 (10H, m, H-3–H-7) ppm; ^13^C NMR (125.68 MHz, CDCl_3_, 25 °C): δ_C_ 98.9 (CH, C-2′), 84.8 (C, C-10), 68.0 (CH, C-11), 67.7 (CH_2_, C-1), 62.3 (CH_2_, C-6′), 30.8 (CH_2_, C-3′), 29.8 (CH_2_, C-2), 29.4 (2 × CH_2_, C-4 and C-5), 29.0 (CH_2_, C-6), 28.7 (CH_2_, C-7), 28.5 (CH_2_, C-8), 26.2 (CH_2_, C-3), 25.5 (CH_2_, C-5′), 19.7 (CH_2_, C-4′) and 18.4 (CH_2_, C-9) ppm.

GC-MS temperature program A *t*_R_ = 15.99 min: *m*/*z* 252.3 (0.1) [M^+^, C_16_H_28_O_2_], 251.2 (0.2), 137.1 (0.2), 135.0 (0.3), 123.1 (0.4), 121.0 (1), 109.0 (3), 107.0 (2), 101.0 (21), 95.0 (12), 93.0 (5), 85.0 (100).

HRESIMS *m*/*z* 275.1945 [M + Na]^+^ (calcd for C_16_H_28_O_2_Na, 275.1982).

#### (22*S*)-1-(2′-Tetrahydropyranyloxy)-22-methyltetracosane-10,19-diyne (9)

**8** (231 mg, 0.91 mmol, 2.5 equiv) was dissolved in dry THF (0.8 mL) and dry HMPA (0.3 mL). *n*-BuLi (0.365 mL, 2.5 mol/L in hexanes, 0.91 mmol, 2.5 equiv) was added slowly at −75 °C, and the mixture was stirred under argon at −40 °C for 2 h. A solution of **6** (100 mg, 0.37 mmol, 1.0 equiv) in dry THF (1.0 mL) was then added slowly to the mixture at −75 °C, and the mixture was allowed to reach r.t over 2 h. TBAI (13.6 mg, 0.037 mmol, 0.1 equiv) was added and the mixture was heated to 80 °C for 18 h, followed by addition of saturated aqueous NH_4_Cl (10 mL) and H_2_O (10 mL). The aqueous phase was separated and extracted with Et_2_O (5 × 20 mL). The organic phases were combined, dried over anhydrous Na_2_SO_4_, filtered, and evaporated under reduced pressure. The product was purified by silica gel flash chromatography (*n*-hexane:EtOAc 98:2 → 9:1) and dried in vacuo to obtain a clear, colorless liquid (140 mg, 85%). *R*_f_ = 0.51 (*n*-hexane:EtOAc 9:1).

^1^H NMR (499.82 MHz, CDCl_3_, 25 °C): δ_H_ 4.57 (1H, dd, ^3^*J*_2′,3′a_ = 2.9 Hz, ^3^*J*_2′,3′b_ = 4.4 Hz, H-2′), 3.87 (1H, ddd, ^2^*J*_6′a,6′b_ = −11.1 Hz, ^3^*J*_6′a,5′b_ = 2.9 Hz, ^3^*J*_6′a,5′a_ = 8.2 Hz, H-6′a), 3.73 (1H, dt, ^2^*J*_1a,1b_ = −9.6 Hz, ^3^*J*_1a,2_ = 6.9 Hz, H-1a), 3.50 (1H, ddd, ^3^*J*_6′b,5′b_ = 3.6 Hz, ^3^*J*_6′b,5′a_ = 7.0 Hz, H-6′b), 3.38 (1H, dt, ^3^*J*_1b,2_ = 6.7 Hz, H-1b), 2.15 (2H, tt, ^5^*J*_9,12_ = 2.5 Hz, ^3^*J*_9,8_ = 6.5 Hz, H-9) 2.14 (2H, ddt, ^5^*J*_18,21a_ = 2.4 Hz, ^5^*J*_18,21b_ = 2.5 Hz, ^3^*J*_18,17_ = 7.2 Hz, H-18), 2.13 (2H, tt, ^3^*J*_12,13_ = 6.8 Hz, H-12), 2.12 (1H, ddt, ^2^*J*_21a,21b_ = −16.4 Hz, ^3^*J*_21a,22_ = 5.8 Hz, H-21a), 2.03 (1H, ddt, ^3^*J*_21b,22_ = 6.9 Hz, H-21b), 1.83 (1H, ddddd, ^2^*J*_4′a,4′b_ = −11.9 Hz, ^3^*J*_4′a,5′b_ = 3.1 Hz, ^3^*J*_4’a,3′b_ = 3.6 Hz, ^3^*J*_4′a,5′a_ = 7.9 Hz, ^3^*J*_4′a,3′a_ = 9.3 Hz, H-4′a), 1.71 (1H, dddd, ^2^*J*_3′a,3′b_ = −12.7 Hz, ^3^*J*_3′a,4′b_ = 4.3 Hz, H-3′a), 1.59 (2H, ddt, ^3^*J*_2,3_ = 7.9 Hz, H-2), 1.58–1.41 (16H, m, H-3′b, H-4′b, H-5′a, H-5′b, H-5, H-6, H-8, H-13, H-17, H-22, H-23a), 1.40–1.25 (6H, m, H-3, H-4, H-7), 1.21 (1H, ddq, ^2^*J*_23b,23a_ = −13.8 Hz, ^3^*J*_23b,24_ = 7.3 Hz, ^3^*J*_23b,22_ = 7.4 Hz, H-23b), 0.95 (3H, d, ^3^*J*_22-CH3,22_ = 6.7 Hz, 22-CH_3_), and 0.88 (3H, dd, ^3^*J*_24,23a_ = 7.6 Hz, H-24) ppm.

^13^C NMR (125.68 MHz, CDCl_3_, 25 °C): δ_C_ 98.9 (CH, C-2′), 81.0 (C, C-19), 80.3 (C, C-11), 80.2 (C, C-10), 79.0 (C, C-20), 67.7 (CH_2_, C-1), 62.4 (CH_2_, C-6′), 34.6 (CH, C-22), 30.8 (CH_2_, C-3′), 29.8 (CH_2_, C-2), 29.5–28.8 (11 × CH_2_, C-4–C-8, C-13–C-17, C-23), 26.2 (CH_2_, C-3), 25.8 (CH_2_, C-21), 25.5 (CH_2_, C-5′), 19.7 (CH_2_, C-4′), 19.1 (CH_3_, 22-CH_3_), 18.8 (3 × CH_2_, C-9, C-12, C-18) and 11.5 (CH_3_, C-24) ppm.

HRESIMS *m*/*z* 467.3944 [M + Na]^+^ (calcd for C_30_H_52_O_2_Na, 467.3860).

#### (22*S*)-22-Methyltetracosane-10,19-diyn-1-ol (10)

**9** (378 mg, 0.85 mmol, 1.0 equiv) was dissolved in dry THF (6 mL) and dry MeOH (12 mL), and CSA (20 mg, 0.085 mmol, 0.1 equiv) was added. The mixture was stirred at r.t. under argon for 16 h and then concentrated under reduced pressure. The product was purified by silica gel flash chromatography (*n*-hexane:EtOAc 9:1 → 2:1) and dried in vacuo to obtain a clear, colorless liquid (249 mg, 81%). *R*_f_ = 0.39 (*n*-hexane:EtOAc 4:1).

^1^H NMR (499.82 MHz, CDCl_3_, 25 °C): δ_H_ 3.64 (2H, dt, ^3^*J*_1,1-OH_ = 4.4 Hz, ^3^*J*_1,2_ = 6.6 Hz, H-1), 2.15 (2H, tt, ^5^*J*_9,12_ = 2.4 Hz, ^3^*J*_9,8_ = 5.5 Hz, H-9), 2.14 (2H, ddt, ^5^*J*_18,21a_ = 2.3 Hz, ^5^*J*_18,21b_ = 2.4 Hz, ^3^*J*_18,17_ = 7.3 Hz, H-18), 2.13 (2H, tt, ^3^*J*_12,13_ = 5.3 Hz, H-12), 2.12 (1H, ddt, ^2^*J*_21a,21b_ = −16.4 Hz, ^3^*J*_21a,22_ = 5.8 Hz, H-21a), 2.03 (1H, ddt, ^3^*J*_21b,22_ = 7.1 Hz, H-21b), 1.57 (2H, tt, ^3^*J*_2,3_ = 7.9 Hz, H-2), 1.52 (1H, ddddq, ^3^*J*_22,22-CH3_ = 6.7 Hz, ^3^*J*_22,23a_ = 7.4 Hz, ^3^*J*_22,23b_ = 7.6 Hz, H-22), 1.50–1.25 (24H, m, H-3–H-8, H-13–H-18, H-23a), 1.23 (1H, d, 1-OH), 1.21 (1H, ddq, ^2^*J*_23b,23a_ = −13.7 Hz, ^3^*J*_23b,24_ = 7.3 Hz, H-23b), 0.95 (3H, d, 22-CH_3_), and 0.88 (3H, dd, ^3^*J*_24,23a_ = 7.6 Hz, H-24) ppm.

^13^C NMR (125.68 MHz, CDCl_3_, 25 °C): δ_C_ 81.0 (C, C-19), 80.3 (C, C-11), 80.2 (C, C-10), 79.0 (C, C-20), 63.1 (CH_2_, C-1), 34.6 (CH, C-22), 32.8 (CH_2_, C-2), 29.5–28.7 (10 × CH_2_, C-5–C-8, C-13–C-17, C-23), 29.4 (CH_2_, C-4), 25.8 (CH_2_, C-21), 25.7 (CH_2_, C-3), 19.1 (CH_3_, 22-CH_3_), 18.8 (3 × CH_2_, C-9, C-12, C-18), and 11.5 (CH_3_, C-24) ppm.

HRESIMS *m*/*z* 361.3462 [M + H]^+^ (calcd for C_25_H_45_O, 361.3465).

#### (22*S*)-22-Methyltetracosan-1-ol (11)

**10** (125 mg, 0.35 mmol, 1.0 mass-eq ) was dissolved in dry EtOAc (7 mL), and Pd/C (10 wt % Pd, 251 mg, 2.0 mass-eq ) was added. The mixture was stirred vigorously in a Parr reactor under H_2_ atmosphere (5 bar) for 5 h and filtered through diatomaceous earth. The diatomaceous earth was washed with Et_2_O (150 mL), and the filtrate was concentrated under reduced pressure. The product was purified by silica gel flash chromatography (*n*-hexane:EtOAc 4:1) and dried in vacuo to obtain a white solid (107 mg, 83%). *R*_f_ = 0.46 (*n*-hexane:EtOAc 4:1).

^1^H NMR (499.82 MHz, CDCl_3_, 25 °C): δ_H_ 3.64 (2H, dt, ^3^*J*_1,1-OH_ = 5.4 Hz, ^3^*J*_1,2_ = 6.6 Hz, H-1), 1.57 (2H, tt, ^3^*J*_2,3_ = 7.6 Hz, H-2), 1.38–1.22 (39H, m, H-3–H-19, H-20a, H-20b, H-21a, H-22, H-23a), 1.21 (1H, t, 1-OH), 1.12 (1H, ddq, ^2^*J*_23b,23a_ = −13.8 Hz, ^3^*J*_23b,24_ = 7.3 Hz, ^3^*J*_23b,22_ = 8.1 Hz, H-23b), 1.07 (1H, dddd, ^2^*J*_21b,21a_ = −13.9 Hz, ^3^*J*_21b,20a_ = 4.2 Hz, ^3^*J*_21b,20b_ = 6.2 Hz, ^3^*J*_21b,22_ = 8.2 Hz, H-21b), 0.85 (3H, dd, ^3^*J*_24,23a_ = 7.3 Hz, H-24), and 0.84 (3H, d, ^3^*J*_22-CH3,22_ = 6.6 Hz, 22-CH_3_) ppm.

^13^C NMR (125.68 MHz, CDCl_3_, 25 °C): δ_C_ 63.1 (CH_2_, C-1), 36.7 (CH_2_, C-21), 34.4 (CH, C-22), 32.8 (CH_2_, C-2), 30.1–29.6 (15 × CH_2_, C-6–C-19, C-23), 29.5 (CH_2_, C-5), 29.4 (CH_2_, C-4), 27.1 (CH_2_, C-20), 25.8 (CH_2_, C-3), 19.2 (CH_3_, 22-CH_3_), and 11.4 (CH_3_, C-24) ppm.

HRESIMS *m*/*z* 391.3846 [M + Na]^+^ (calcd for C_25_H_52_ONa, 391.3910).

#### (22*S*)-22-Methyltetracosanoic Acid (12)

**11** (72 mg, 0.20 mmol, 1.0 equiv) was dissolved in THF (2 mL), acetone (6 mL), and EtOAc (2 mL), and Jones reagent (2.0 mol/L CrO_3_ in aq. H_2_SO_4_, 225 μL, 0.45 mmol, 2.3 equiv) was added dropwise. The mixture was stirred at r.t. for 1.5 h, quenched with 2-propanol (3 mL), and filtered through diatomaceous earth. The diatomaceous earth was washed with Et_2_O (200 mL), and the filtrate was concentrated under reduced pressure. The product was purified by silica gel flash chromatography (*n*-hexane:EtOAc:AcOH 9:1:0.01) and dried in vacuo to obtain a white solid (71 mg, 95%).  + 4.7 (*c* 0.48, CHCl_3_); lit.  + 4.3 (*c* 0.67, CHCl_3_);^35^*R*_f_ = 0.61 (*n*-hexane:EtOAc:AcOH 7:3:0.01).

^1^H NMR (499.82 MHz, CDCl_3_, 25 °C): δ_H_ 2.35 (2H, t, ^3^*J*_2,3_ = 7.5 Hz, H-2), 1.63 (2H, tt, ^3^*J*_3,4_ = 7.6 Hz, H-3), 1.38–1.21 (37H, m, H-4–H-19, H-20a, H-20b, H-21a, H-22, H-23a), 1.12 (1H, ddq, ^2^*J*_23b,23a_ = −13.9 Hz, ^3^*J*_23b,24_ = 7.3 Hz, ^3^*J*_23b,22_ = 8.1 Hz, H-23b), 1.07 (1H, dddd, ^2^*J*_21b,21a_ = −13.9 Hz, ^3^*J*_21b,20a_ = 2.2 Hz, ^3^*J*_21b,20b_ = 6.1 Hz, ^3^*J*_21b,22_ = 8.3 Hz, H-21b), 0.85 (3H, dd, ^3^*J*_24,23a_ = 7.3 Hz, H-24), and 0.84 (3H, d, ^3^*J*_22-CH3,22_ = 6.6 Hz, 22-CH_3_) ppm.

^13^C NMR (125.68 MHz, CDCl_3_, 25 °C): δ_C_ 179.2 (C, C-1), 36.7 (CH_2_, C-21), 34.4 (CH, C-22), 33.9 (CH_2_, C-2), 30.1–29.4 (15 × CH_2_, C-6–C-19, C-23), 29.3 (CH_2_, C-5), 29.1 (CH_2_, C-4), 27.1 (CH_2_, C-20), 24.7 (CH_2_, C-3), 19.2 (CH_3_, 22-CH_3_), and 11.4 (CH_3_, C-24) ppm.

HRESIMS *m*/*z* 405.3786 [M + Na]^+^ (calcd for C_25_H_50_O_2_Na, 405.3703).

#### (22*S*)-22-Methyltetracosanyl Oleate (*anteiso*-C_25:0_/C_18:1_ WE; 13)

**11** (54 mg, 0.15 mmol, 1.0 equiv) was dissolved in dry CH_2_Cl_2_ (4.5 mL) and dry pyridine (1.8 mL), and DMAP (18 mg, 0.15 mmol, 1.0 equiv) and EDC·HCl (56 mg, 0.29 mmol, 2.0 equiv) were added. A solution of oleic acid (50 mg, 0.18 mmol, 1.2 equiv) in dry CH_2_Cl_2_ (0.9 mL) was added dropwise to the reaction mixture, which then was stirred at r.t. under argon for 40 h and diluted with CH_2_Cl_2_ (10 mL). The reaction mixture was washed with aqueous HCl (1 mol/L, 2 × 10 mL), and the combined aqueous phases were extracted with CH_2_Cl_2_ (3 × 10 mL). The organic phases were combined, dried over anhydrous Na_2_SO_4_, filtered, and evaporated under reduced pressure. The product was purified by silica gel flash chromatography (*n*-hexane:EtOAc 95:5) and dried in vacuo to obtain a white solid (80 mg, 87%). Mp = 27.7 °C;  + 0.9 (*c* 1.80, CHCl_3_); *R*_f_ = 0.41 (*n*-hexane:EtOAc 95:5).

^1^H NMR (499.82 MHz, CDCl_3_, 25 °C): δ_H_ 5.35 (1H, dtt, ^4^*J*_9′,11′_ = −1.5 Hz, ^3^*J*_9′,8′_ = 7.2 Hz, ^3^*J*_9′,10′_ = 10.9 Hz, H-9′), 5.34 (1H, dtt, ^4^*J*_10′,8′_ = −1.5 Hz, ^3^*J*_10′,11′_ = 7.2 Hz, H-10′), 4.05 (2H, t, ^3^*J*_1,2_ = 6.7 Hz, H-1), 2.27 (2H, t, ^3^*J*_2′,3′_ = 7.6 Hz, H-2′), 2.01 (2H, ddt, ^3^*J*_11′,12′_ = 7.0 Hz, H-11′), 2.00 (2H, ddt, ^3^*J*_8′,7′_ = 7.3 Hz, H-8′), 1.61 (2H, tt, ^3^*J*_3′,4′_ = 7.2 Hz, H-3′), 1.61 (2H, tt, ^3^*J*_2,3_ = 7.4 Hz, H-2), 1.37–1.21 (59H, m, H-3–H-19, H-4′–H-7′, H-12′–H-17′, H-20a, H-20b, H-21a, H-22, H23-a), 1.12 (1H, ddq, ^2^*J*_23b,23a_ = −13.7 Hz, ^3^*J*_23b,24_ = 7.3 Hz, ^3^*J*_23b,22_ = 7.8 Hz, H-23b), 1.07 (1H, dddd, ^2^*J*_21b,21a_ = −13.9 Hz, ^3^*J*_21b,20a_ = 4.3 Hz, ^3^*J*_21b,20b_ = 6.1 Hz, ^3^*J*_21b,22_ = 8.2 Hz, H-21b), 0.88 (3H, t, ^3^*J*_18′,17′_ = 7.0 Hz, H-18′), 0.85 (3H, dd, ^3^*J*_24,23a_ = 7.4 Hz, H-24) and 0.84 (3H, d, ^3^*J*_22-CH3,22_ = 6.7 Hz, 22-CH_3_) ppm.

^13^C NMR (125.68 MHz, CDCl_3_, 25 °C): δ_C_ 174.0 (C, C-1′), 130.0 (C, C-9′), 129.8 (C, C-10′), 64.4 (CH_2_, C-1), 36.7 (CH_2_, C-21), 34.4 (CH_2_ and CH, C-2′, C-22), 31.9 (CH_2_, C-16′), 30.1–29.1 (25 × CH_2_, C-4–C-19, C-4′–C-7′, C-12′–C-15′, C-23), 28.7 (CH_2_, C-2), 27.2 (3 × CH_2_, C-8′, C-11′, C-20), 26.0 (CH_2_, C-3), 25.0 (CH_2_, C-3′), 22.7 (CH_2_, C-17′), 19.2 (CH_3_, 22-CH_3_), 14.1 (CH_3_, C-18′) and 11.4 (CH_3_, C-24) ppm.

HRESIMS *m*/*z* 633.6570 [M + H]^+^ (calcd for C_43_H_85_O_2_, 633.6544).

#### Cholesteryl (22′*S*)-22′-Methyltetracosanoate (*anteiso*-C_25:0_ CE; 14)

**12** (28 mg, 0.073 mmol, 1.0 equiv) was dissolved in dry toluene (2 mL), and dry DMF (0.57 μL, 7.3 μmol, 0.1 equiv) and SOCl_2_ (27 μL, 0.367 mmol, 5.0 equiv) were added, which resulted in bubbling of the solution. The solution was stirred at 70 °C for 3 h and then concentrated under reduced pressure. To the crude acyl chloride was slowly added a solution of cholesterol (34 mg, 0.088 mmol, 1.2 equiv), DMAP (12 mg, 0.102 mmol, 1.4 equiv), and dry Et_3_N (24 μL, 0.176 mmol, 2.4 equiv) in dry toluene (1.0 mL) at 0 °C. The mixture was stirred at r.t. under argon for 1.5 h, and then diluted with CHCl_3_ (10 mL) and saturated aqueous NaHCO_3_ (10 mL). The aqueous phase was extracted with CHCl_3_ (3 × 10 mL). The organic phases were combined, dried over anhydrous Na_2_SO_4_, filtered, and evaporated under reduced pressure. The product was purified by silica gel flash chromatography (*n*-hexane:EtOAc 9:1) and dried in vacuo to obtain a white solid (34 mg, 62%). Mp = 82.5 °C;  – 15.0 (*c* 2.90, CHCl_3_); *R*_f_ = 0.69 (*n*-hexane:EtOAc 9:1).

^1^H NMR (499.82 MHz, CDCl_3_, 25 °C): δ_H_ 5.37 (1H, dddd, ^4^*J*_6,4b_ = −1.5 Hz, ^4^*J*_6,4a_ = −0.4 Hz, ^3^*J*_6,7b_ = 2.2 Hz, ^3^*J*_6,7a_ = 5.0 Hz, H-6), 4.61 (1H, dddd, ^3^*J*_3,2a_ = 4.3 Hz, ^3^*J*_3,4a_ = 5.0 Hz, ^3^*J*_3,4b_ = 11.4 Hz, ^3^*J*_3,2b_ = 11.8 Hz, H-3), 2.32 (1H, ddddd, ^2^*J*_4a,4b_ = −14.2 Hz, ^5^*J*_4a,7b_ = 0.2 Hz, ^5^*J*_4a,7a_ = 0.8 Hz, H-4a), 2.30 (1H, ddddd, ^5^*J*_4b,7a_ = 2.3 Hz, ^5^*J*_4b,7b_ = 2.7 Hz, H-4b), 2.26 (2H, t, ^3^*J*_2′,3′_ = 7.5 Hz, H-2′), 2.01 (1H, ddd, ^2^*J*_12a,12b_ = −12.8 Hz, ^3^*J*_12a,11a_ = 3.1 Hz, ^3^*J*_12a,11b_ = 4.3 Hz, H-12a), 1.97 (1H, ddddd, ^2^*J*_7a,7b_ = −17.7 Hz, ^3^*J*_7a,8_ = 6.2 Hz, H-7a), 1.86 (1H, ddd, ^2^*J*_1a,1b_ = −13.4 Hz, ^3^*J*_1a,2a_ = 3.6 Hz, ^3^*J*_1a,2b_ = 3.7 Hz, H-1a), 1.85 (1H, dddd, ^2^*J*_2a,2b_ = −12.2 Hz, ^3^*J*_2a,1b_ = 3.4 Hz, H-2a), 1.83 (1H, dddd, ^2^*J*_16a,16b_ = −13.4 Hz, ^3^*J*_16a,15b_ = 6.2 Hz, ^3^*J*_16a,15a_ = 9.6 Hz, ^3^*J*_16a,17_ = 9.7 Hz, H-16a), 1.61 (2H, tt, ^3^*J*_3′,4′_ = 7.6 Hz, H-3′), 1.82–1.42 (7H, m, H-2b, H-7b, H-8, H-11a, H-11b, H-15a, H-25), 1.41–1.21 (41H, m, H-4′–H-19′, H-16b, H-20, H-20′a, H-20′b, H-21′a, H-22a, H-22′, H-23a, H-23′a), 1.20–1.10 (6H, m, H-1b, H-12b, H-23b, H-23′b, H-24a, H-24b), 1.09 (1H, ddd, ^3^*J*_17,20_ = 9.2 Hz, ^3^*J*_17,16b_ = 9.5 Hz, H-17), 1.07 (1H, dddd, ^2^*J*_21′b,21′a_ = −13.9 Hz, ^3^*J*_21′b,20′a_ = 4.3 Hz, ^3^*J*_21′b,20′b_ = 6.1 Hz, ^3^*J*_21′b,22′_ = 8.2 Hz, H-21′b), 1.04 (1H, dddd, ^2^*J*_15b,15a_ = −13.8 Hz, ^3^*J*_15b,16b_ = 5.2 Hz, ^3^*J*_15b,14_ = 12.6 Hz, H-15b), 1.02 (3H, s, H-19), 1.00 (1H, dddd, ^2^*J*_22b,22a_ = −13.6 Hz, ^3^*J*_22b,23a_ = 4.8 Hz, ^3^*J*_22b,20_ = 9.0 Hz, ^3^*J*_22b,23b_ = 9.0 Hz, H-22b), 1.00 (1H, ddd, ^3^*J*_14,15a_ = 7.1 Hz, ^3^*J*_14,8_ = 10.3 Hz, H-14), 0.95 (1H, ddd, ^3^*J*_9,11a_ = 3.8 Hz, ^3^*J*_9,11b_ = 10.3 Hz, ^3^*J*_9,8_ = 11.7 Hz, H-9), 0.91 (3H, d, ^3^*J*_21,20_ = 6.7 Hz, H-21), 0.87 (3H, d, ^3^*J*_26,25_ = 6.6 Hz, H-26), 0.86 (3H, d, ^3^*J*_27,25_ = 6.6 Hz, H-27), 0.85 (3H, dd, ^3^*J*_24′,23′b;24′,23′a_ = 7.3 Hz, H-24′), 0.84 (3H, d, ^3^*J*_22′-CH_3_,22′_ = 6.6 Hz, 22′-CH_3_) and 0.68 (3H, s, H-18) ppm.

^13^C NMR (125.68 MHz, CDCl_3_, 25 °C): δ_C_ 173.3 (C, C-1′), 139.7 (C, C-5), 122.6 (CH, C-3), 73.7 (CH, C-6), 56.7 (CH, C-14), 56.2 (CH, C-17), 50.0 (CH, C-9), 42.3 (C, C-13), 39.8 (CH_2_, C-12), 39.5 (CH_2_, C-24), 38.2 (CH_2_, C-4), 37.0 (CH_2_, C-1), 36.7 (CH_2_, C-21′), 36.6 (C, C-10), 36.3 (CH_2_, C-22), 35.8 (CH, C-20), 34.7 (CH_2_, C-2′), 34.4 (CH, C-22′), 31.9 (CH_2_ and CH, C-7, C-8), 30.1–29.5 (15 × CH_2_, C-6′–C-19′, C-23′), 29.3 (CH_2_, C-5′), 29.1 (CH_2_, C-4′), 28.2 (CH_2_, C-16), 28.0 (CH, C-25), 27.8 (CH_2_, C-2), 27.1 (CH_2_, C-20′), 25.1 (CH_2_, C-3′), 24.3 (CH_2_, C-15), 23.8 (CH_2_, C-23), 22.8–22.6 (2 × CH_3_, C-26, C-27), 21.0 (CH_2_, C-11), 19.3 (CH_3_, C-19), 19.2 (CH_3_, 22′-CH_3_), 18.7 (CH_3_, C-21), 11.9 (CH_3_, C-18), and 11.4 (CH_3_, C-24′) ppm.

HRMS (MALDI) *m*/*z* 773.7392 [M + Na]^+^ (calcd for C_52_H_94_O_2_Na, 773.7146).

### Langmuir–Blodgett Trough Studies

The Langmuir–Blodgett trough studies were performed with a Langmuir trough (KSV NIMA Langmuir large trough, Biolin Scientific; dimensions 580 × 145 mm^2^) equipped with a temperature sensor, surface balance, surface potential meter (KSV SPOT) and a Brewster angle microscope (KSV NIMA microBAM). The trough was filled with a PBS buffer (140 mM NaCl, 3 mM KCl, 10 mM phosphate buffer, pH 7.4) and the lipids dissolved in chloroform (4–5 mM concentration) were spread to the subphase surface with a Hamilton syringe. The chloroform was evaporated for 5 min before starting measurements. The temperature was controlled with a circulating water bath (LAUDA ECO E4) and maintained at 35 ± 1 °C during the measurements. The films were compressed at a constant rate of 10 mm/min (3.7%/min from the initial area). To prevent lipid oxidation by ambient ozone,^57,58^ the measurement setup was kept in an acrylic box (volume 290 L) and dry air was continuously passed through an ODS-3P ozone destruct unit (Hull) into the enclosure at a rate of 76 L/min. All measurements were repeated at least three times.

### WAXS and SAXS Measurements

The WAXS experiments were performed at the X-ray Laboratory, University of Helsinki, using a custom-built X-ray scattering setup. The measurements were conducted in perpendicular transmission geometry mode with a copper X-ray tube, operated with current and voltage of 25 mA and 36 kV, respectively. A Montel-mirror monochromator (Incoated) was applied to select the Cu Kα radiation (wavelength, λ = 1.541 Å). The scattered intensities were collected onto an image plate detector (MAR345, Marresearch).

The length of the scattering vector (*q*)-scale was calibrated for the small (*q* < 1.0 1/Å) and larger (*q* ≥ 1.0 1/Å) scattering angles using powder samples of silver behenate (AgC_22_H_43_O_2_), and lanthanum hexaboride (LaB_6_), respectively. The samples of *anteiso*-C_25:0_/C_18:1_ WE and *anteiso*-C_25:0_ CE were measured as dry powder sealed between two mylar foils in a washer. The measurements were conducted at standard room temperature and humidity.

SAXS and WAXS data were collected at the ForMAX beamline of the MAX IV Synchrotron in Lund, Sweden. Sample preparation and conditions were identical to the laboratory WAXS measurements. A beam energy of 15 keV was used and an EIGER2 X 4 M and a custom Lambda 3 M detector were used to collect SAXS and WAXS, respectively. The distance between sample and detector was approximately 1.7m for SAXS and 0.12m for WAXS.

The analysis of the collected WAXS and SAXS data was conducted using Matlab and Python. For the scattering data, absorption and geometrical corrections were applied and the air scattering was deducted. From the integrated data, the observed peaks with the corresponding *d-spacing* (*d* = 2π/*q*) values were calculated.

## Data Availability

The raw ^1^H and ^13^C NMR data for the end products and synthetic intermediates is available in the SI.

## Supporting Information Available

^1^H and ^13^C NMR spectra for all reported compounds and supporting data related to biophysical studies (PDF)
Raw Bruker ^1^H and ^13^C NMR data files and supporting data related to the biophysical studies (ZIP)
