# Supplementary material for: Another Brick in the Wall of Tear Film Insights Added Through the Total Synthesis and Biophysical Profiling of anteiso-Branched Wax and Cholesteryl Esters
Source: J Nat Prod. 2024 Mar 28;87(4):954–65. doi: 10.1021/acs.jnatprod.3c01247 (PMC11389978; doi:10.1021/acs.jnatprod.3c01247)
Supplement: Supplementary file 1 — np3c01247_si_001.pdf [file np3c01247_si_001.pdf]

Supporting Information

Another Brick in the Wall of Tear Film Insights Added Through the Total Synthesis and Biophysical Profiling of *anteiso*-Branched Wax and Cholesteryl Esters

Henrik Stubb,[1] Tuomo Viitaja,[1,2] Ryan M. Trevorah,[3] Jan-Erik Raitanen,[1] Jukka Moilanen,[2] Kirsi J. Svedström[3] and Filip S. Ekholm[,1]*

1 Department of Chemistry, University of Helsinki, P.O. Box 55, FI-00014 Helsinki, Finland

2 Ophthalmology, University of Helsinki and Helsinki University Hospital, Haartmaninkatu 8, FI-00290 Helsinki, Finland

3 Department of Physics, University of Helsinki, P.O. Box 64, FI-00014 Helsinki, Finland

[*]filip.ekholm@helsinki.fi

Table of Contents

1. NMR Spectra of Synthesized Compounds S3

2. Additional Biophysical Data...S15

1. NMR Spectra of Synthesized Compounds

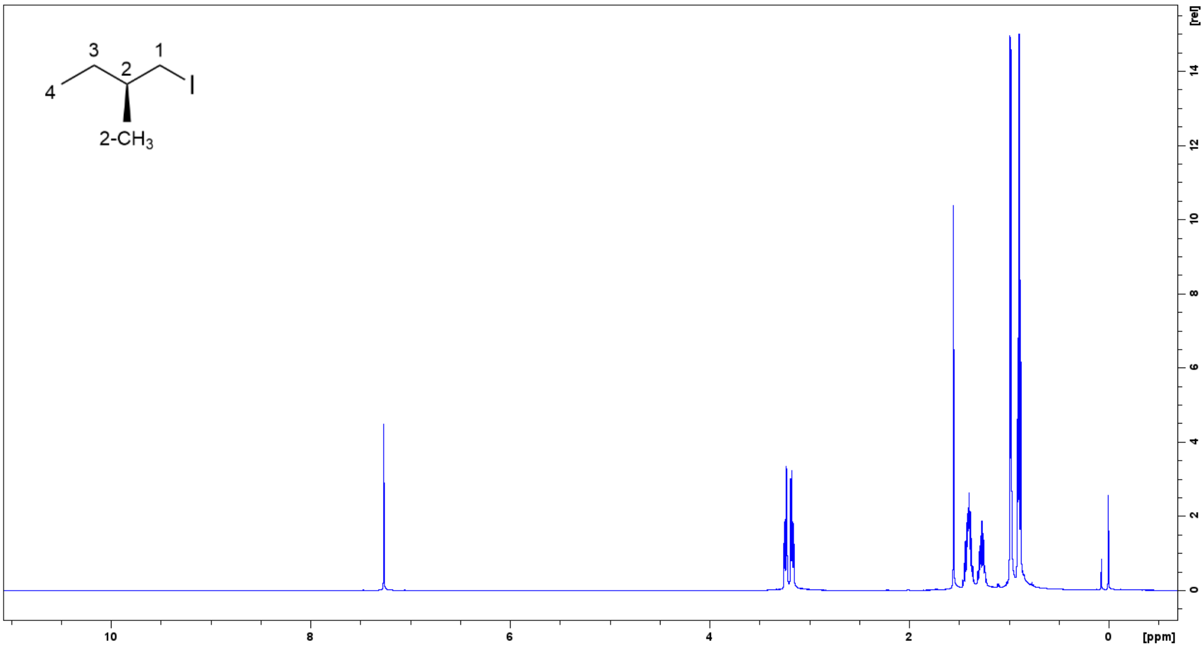

Figure S1. ^{1}H NMR (499.82 MHz) of **1** at 25 °C.

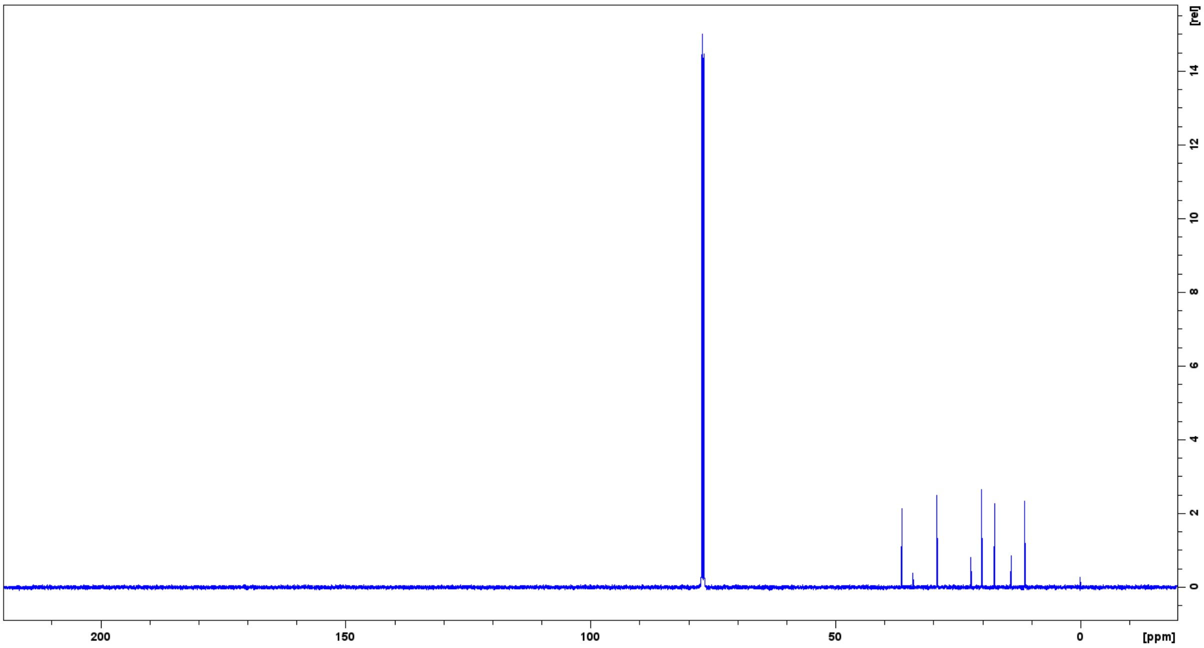

Figure S2. ^{13}C NMR (125.68 MHz) of **1** at 25 °C.

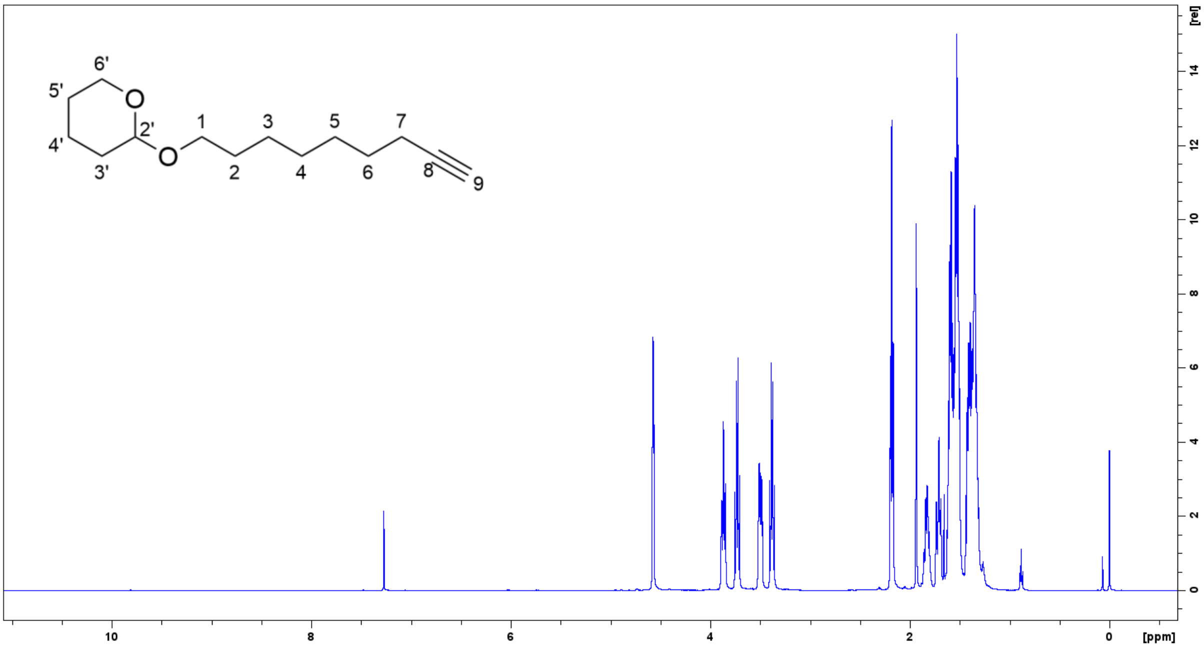

Figure S3. ^{1}H NMR (499.82 MHz) of **3** at 25 °C

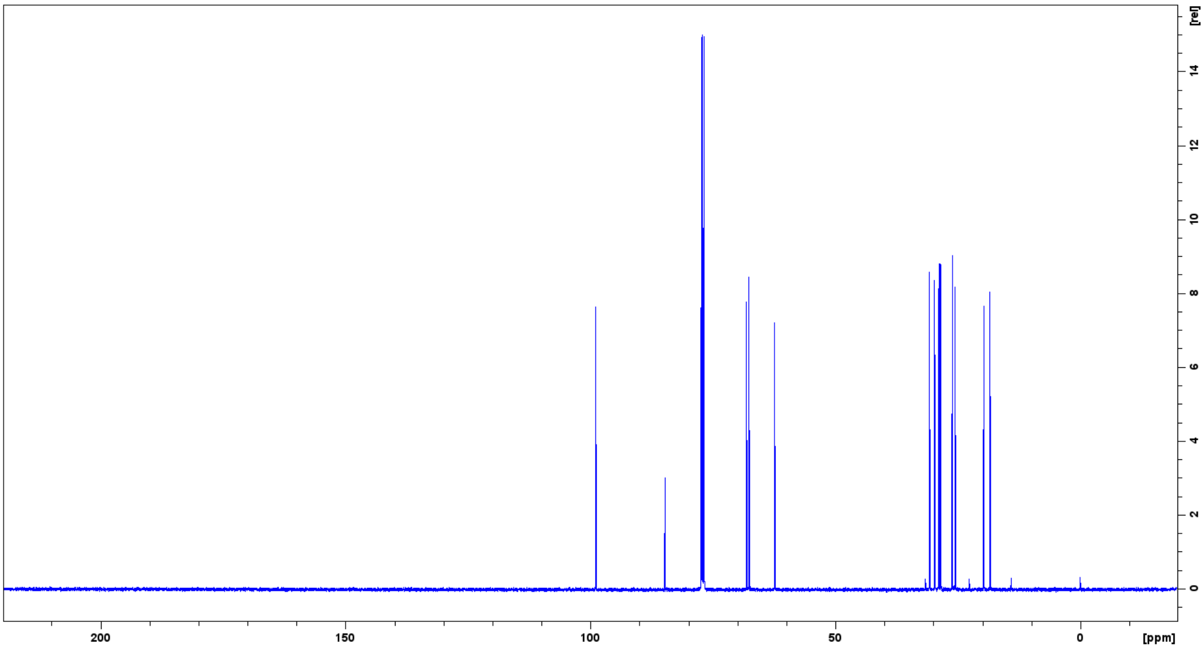

Figure S4. ^{13}C NMR (125.68 MHz) of **3** at 25 °C.

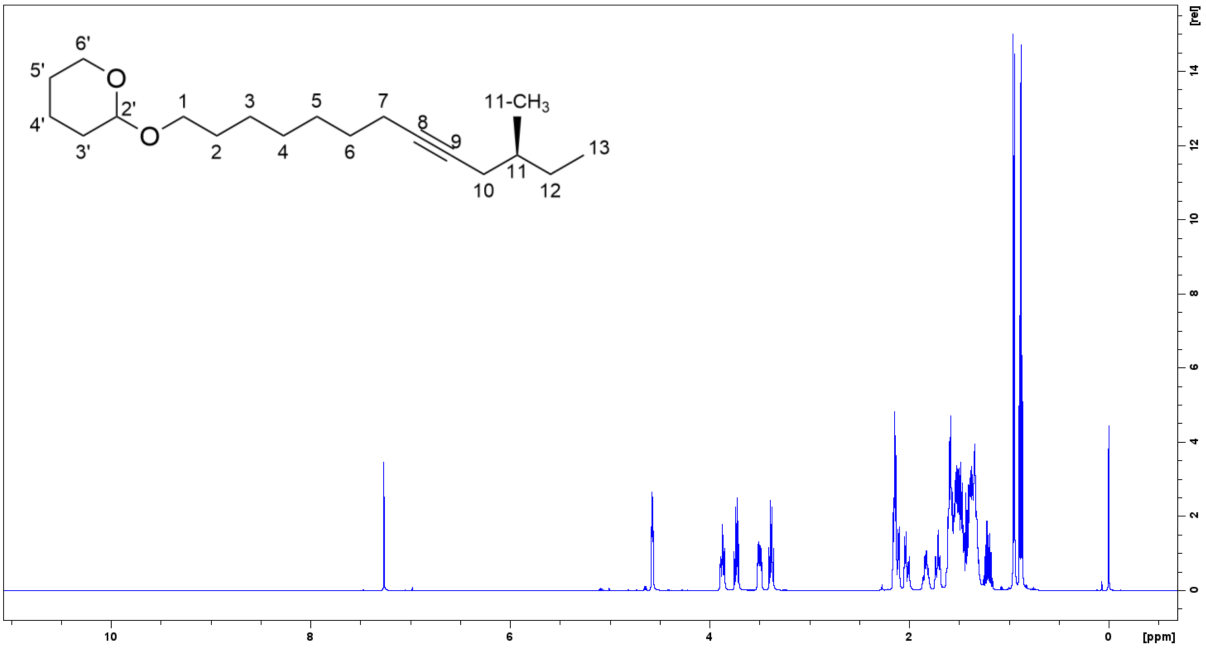

Figure S5. ^{1}H NMR (499.82 MHz) of **4** at 25 °C.

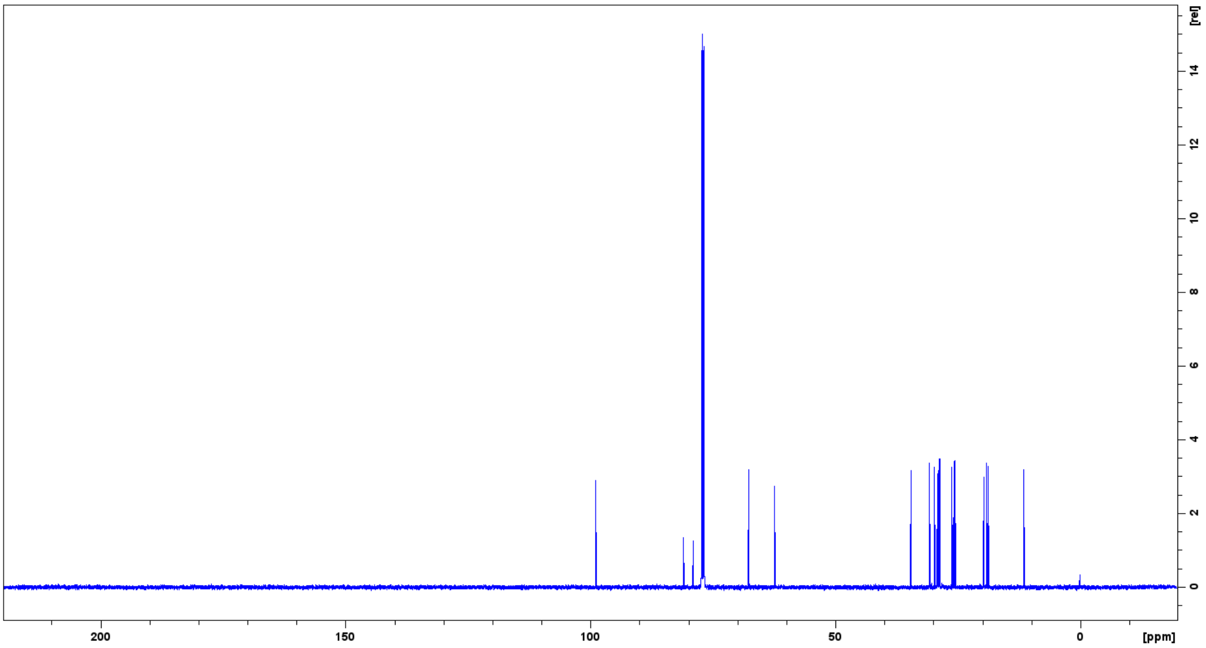

Figure S6. ^{13}C NMR (125.68 MHz) of **4** at 25 °C.

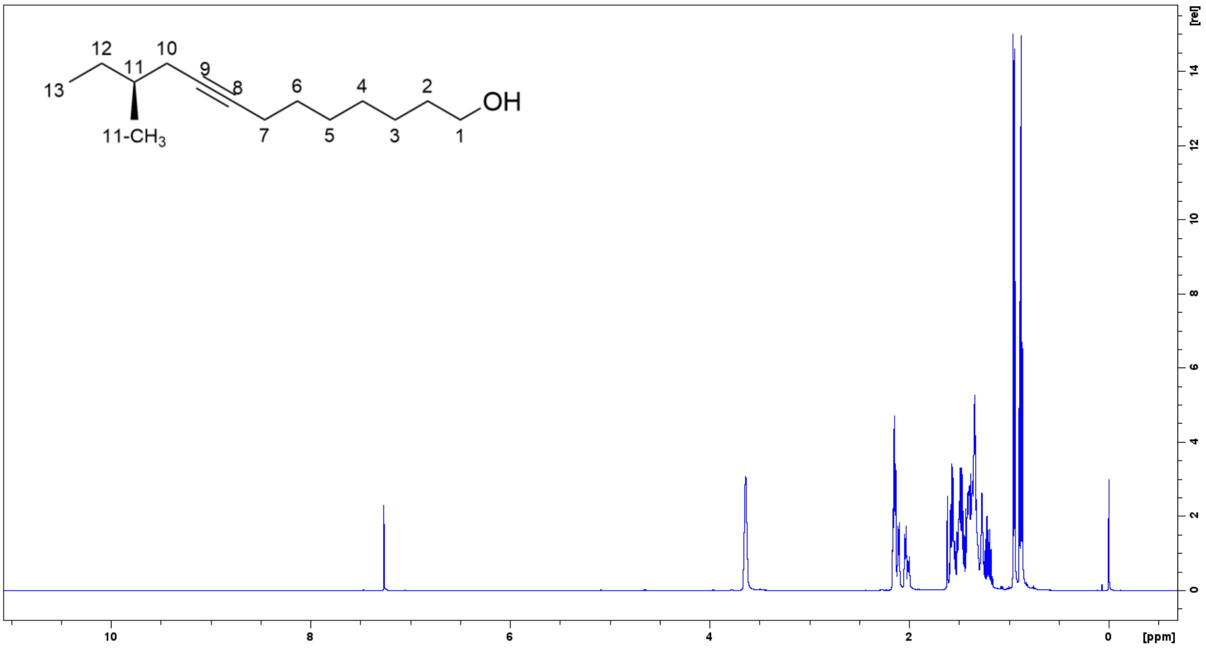

Figure S7. ^{1}H NMR (499.82 MHz) of **5** at 25 °C.

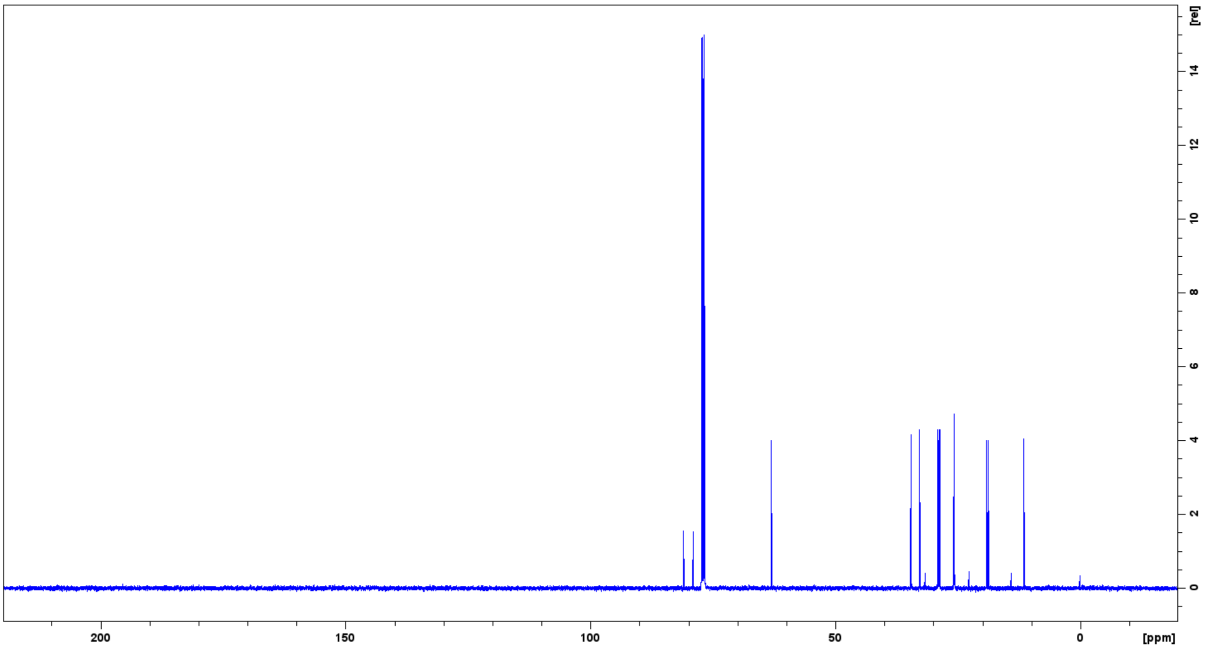

Figure S8. ^{13}C NMR (125.68 MHz) of **5** at 25 °C.

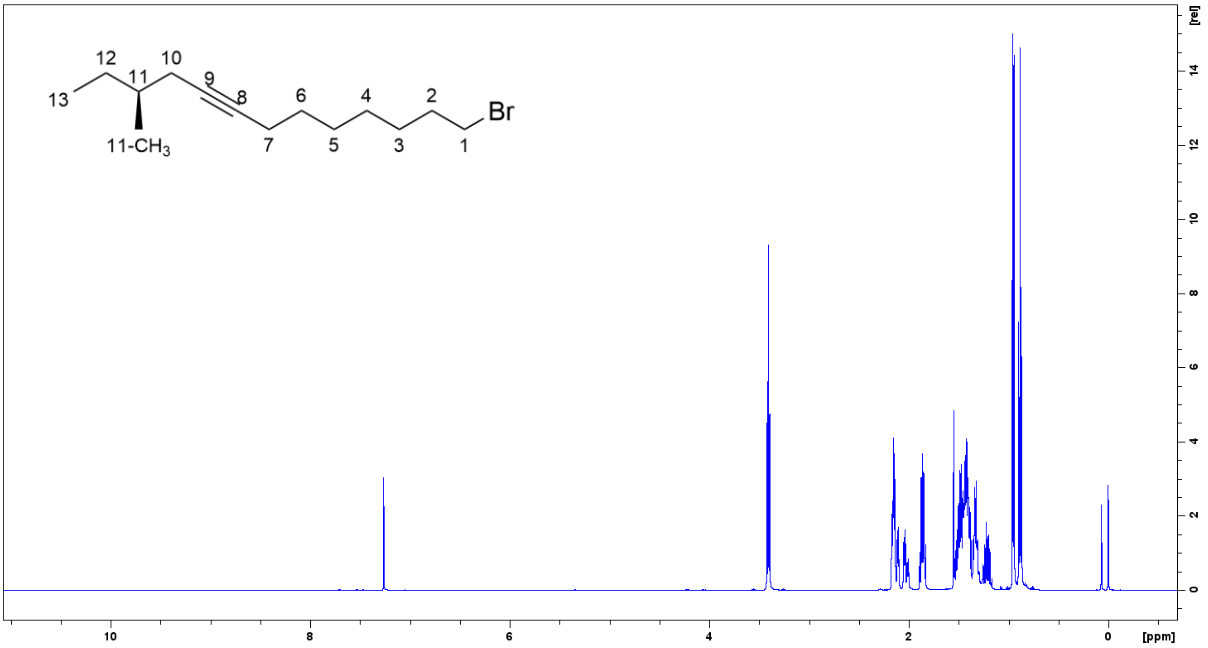

Figure S9. ^{1}H NMR (499.82 MHz) of **6** at 25 °C.

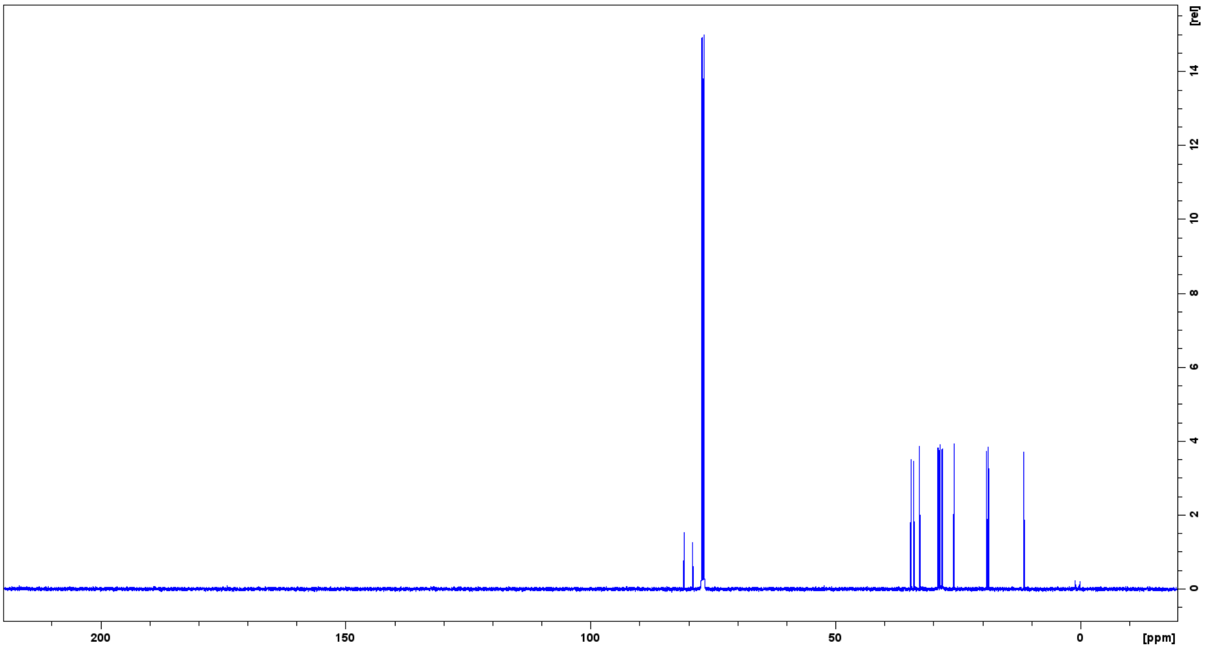

Figure S10. ^{13}C NMR (125.68 MHz) of **6** at 25 °C.

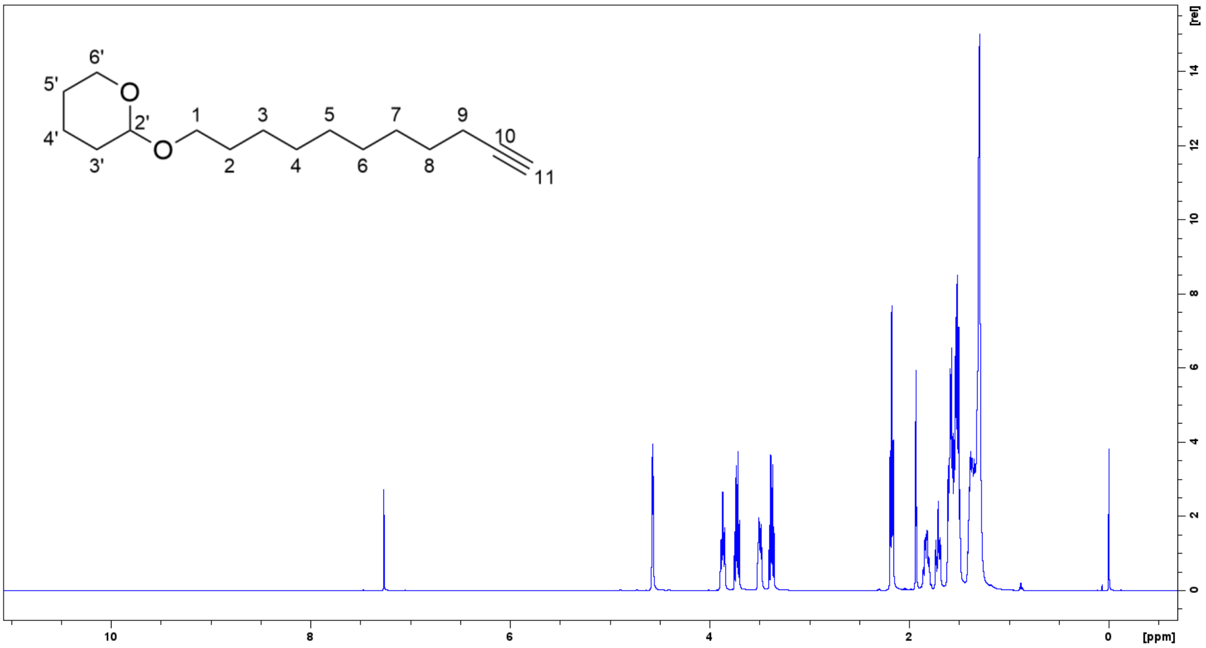

Figure S11. ^{1}H NMR (499.82 MHz) of **8** at 25 °C.

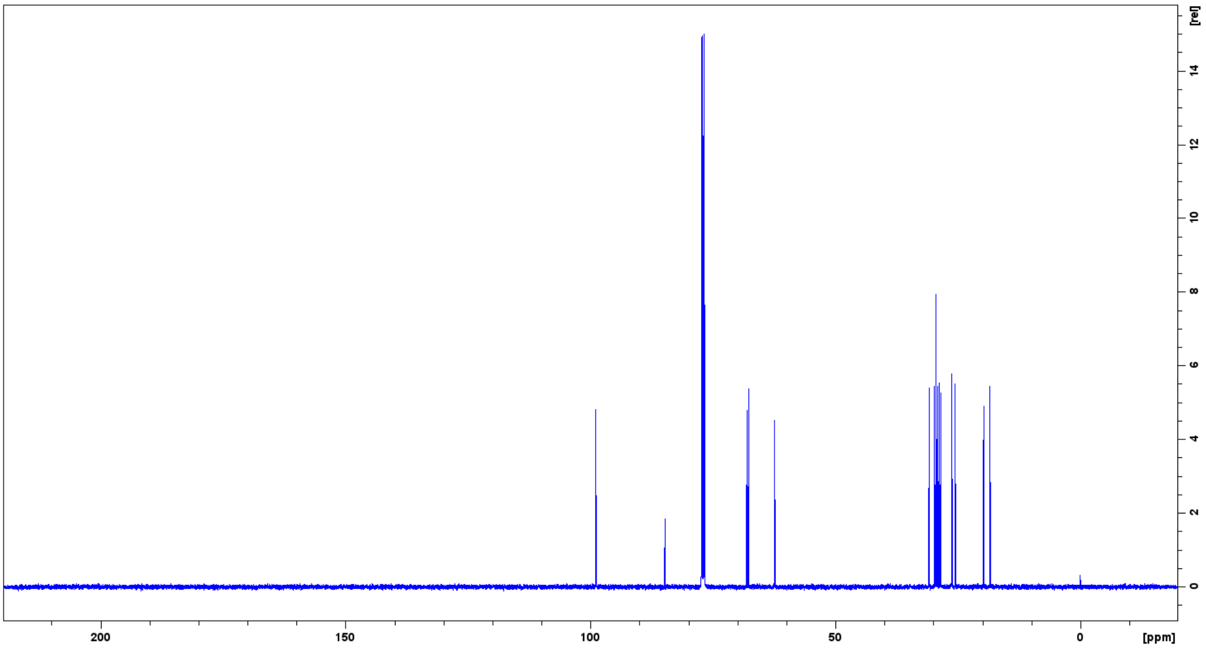

Figure S12. ^{13}C NMR (125.68 MHz) of **8** at 25 °C.

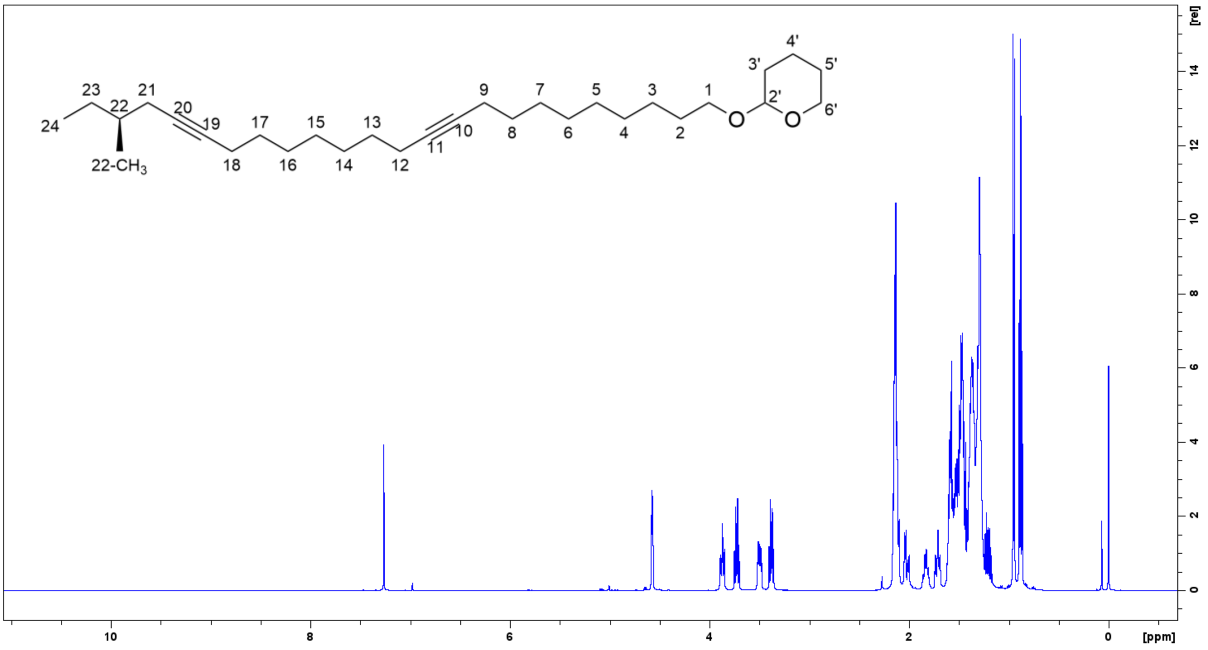

Figure S13. ^{1}H NMR (499.82 MHz) of **9** at 25 °C.

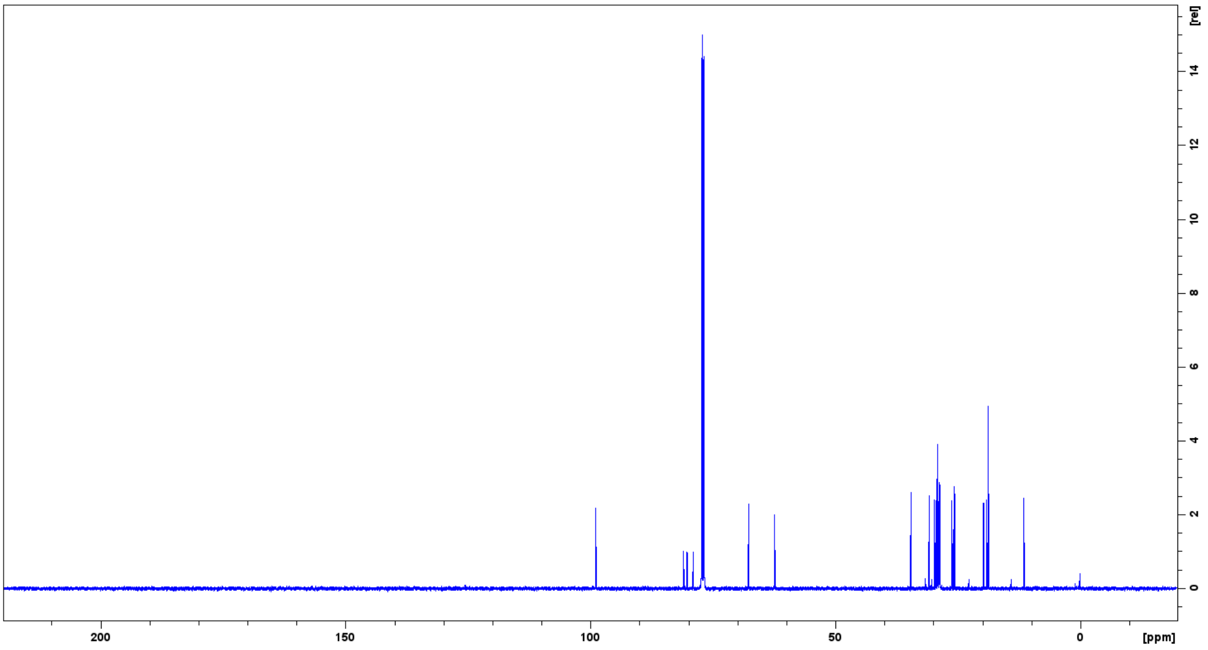

Figure S14. ^{13}C NMR (125.68 MHz) of **9** at 25 °C.

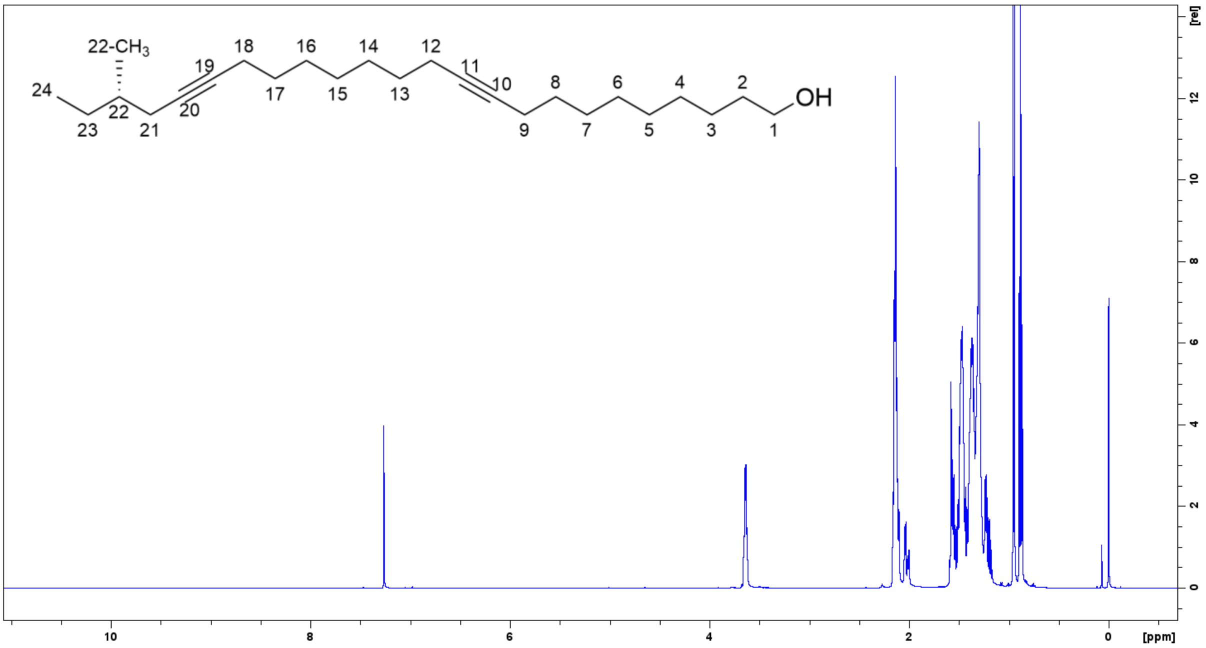

Figure S15. ^{1}H NMR (499.82 MHz) of **10** at 25 °C.

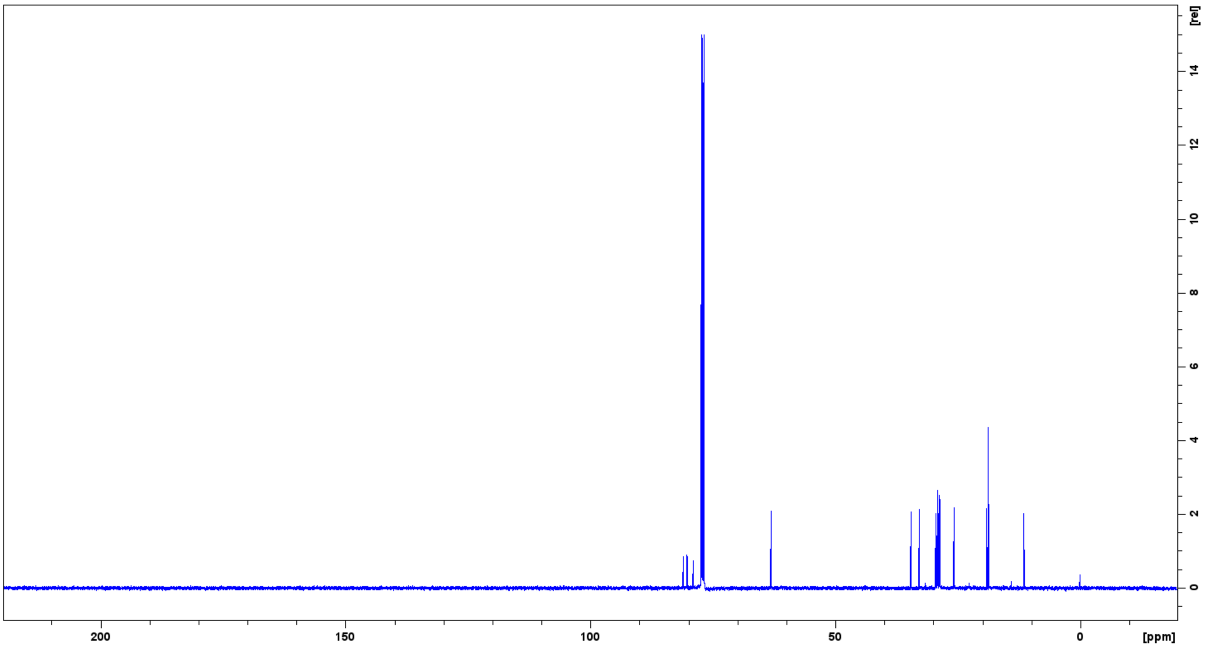

Figure S16. ^{13}C NMR (125.68 MHz) of **10** at 25 °C.

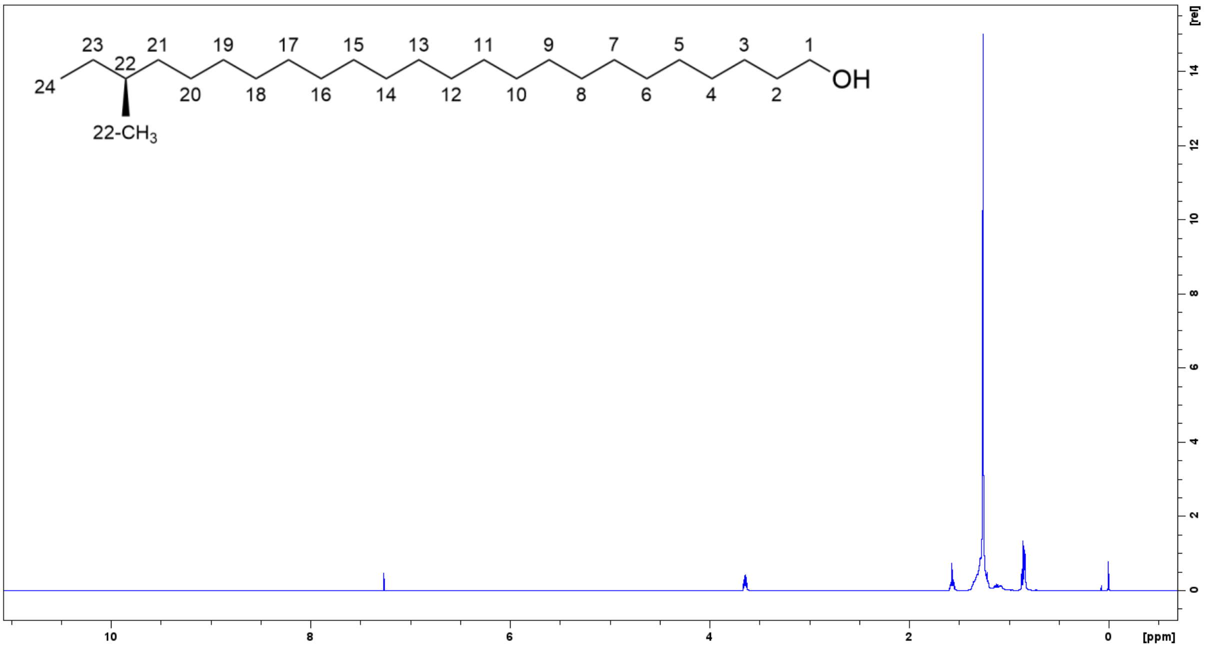

Figure S17. ^{1}H NMR (499.82 MHz) of **11** at 25 °C.

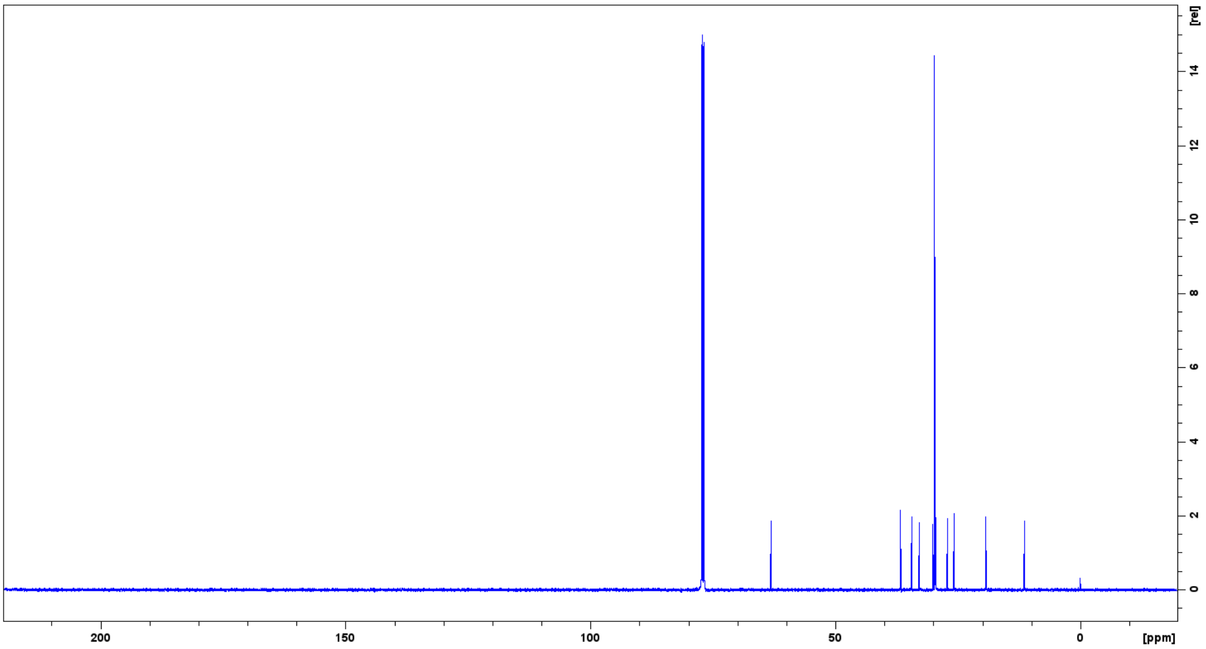

Figure S18. ^{13}C NMR (125.68 MHz) of **11** at 25 °C.

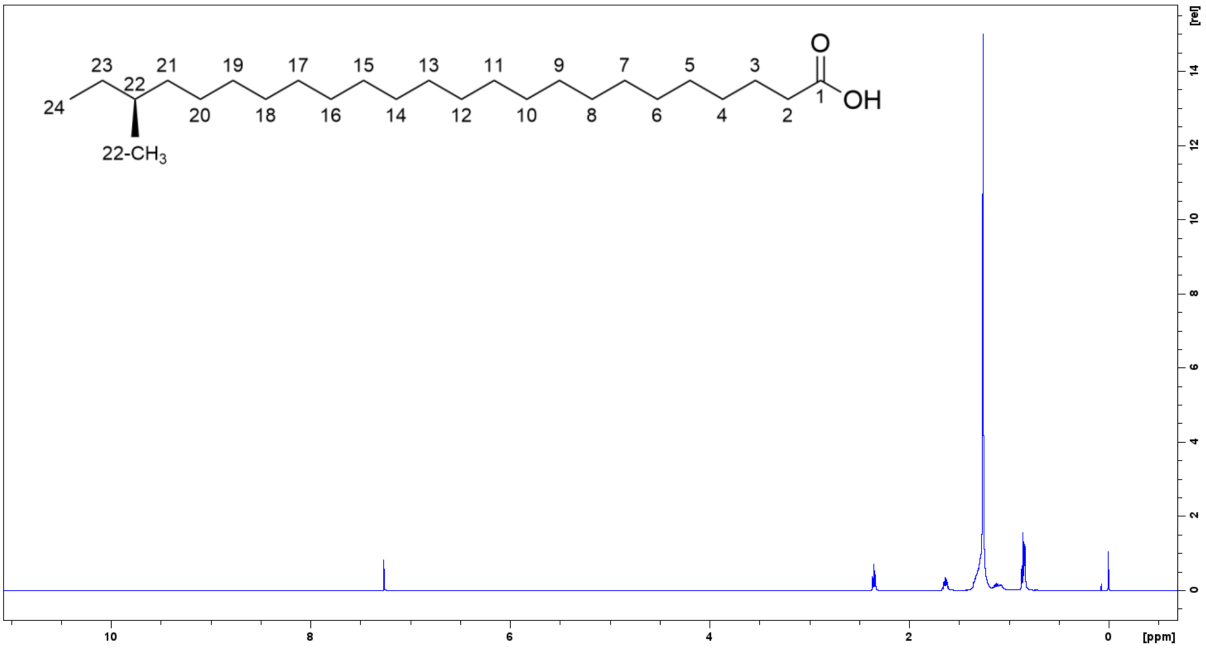

Figure S19. ^{1}H NMR (499.82 MHz) of **12** at 25 °C.

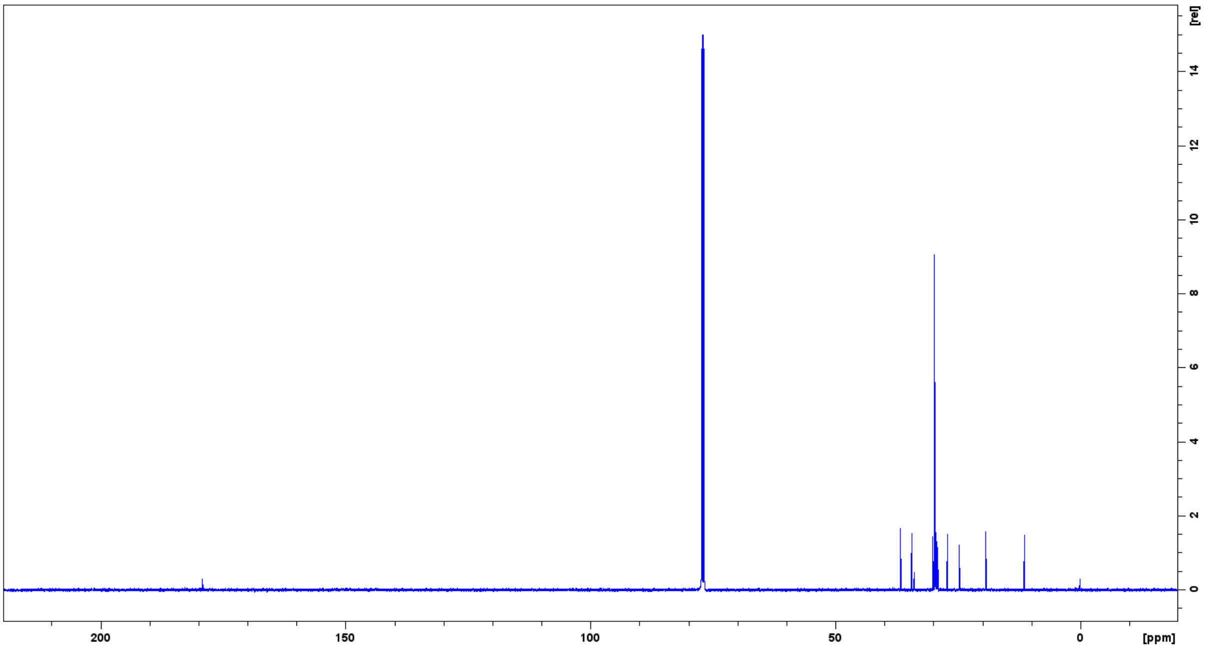

Figure S20. ^{13}C NMR (125.68 MHz) of **12** at 25 °C.

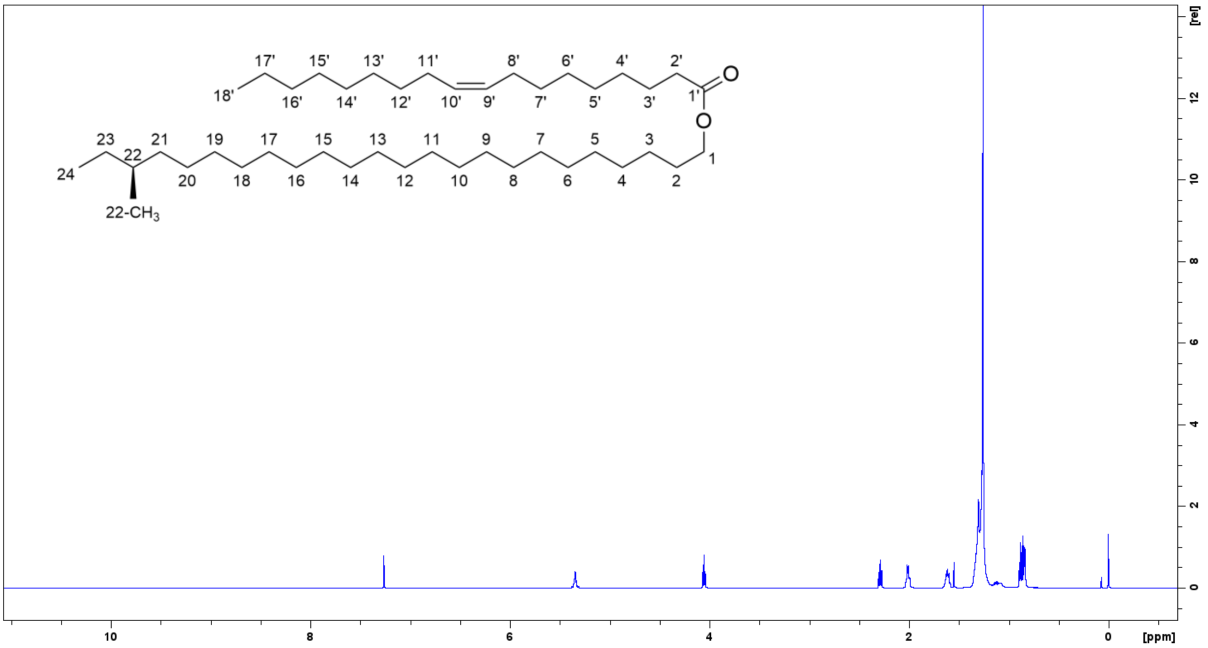

Figure S21. ^{1}H NMR (499.82 MHz) of *anteiso*-$C_{25:0}$/$C_{18:1}$ WE (**13**) at 25 °C.

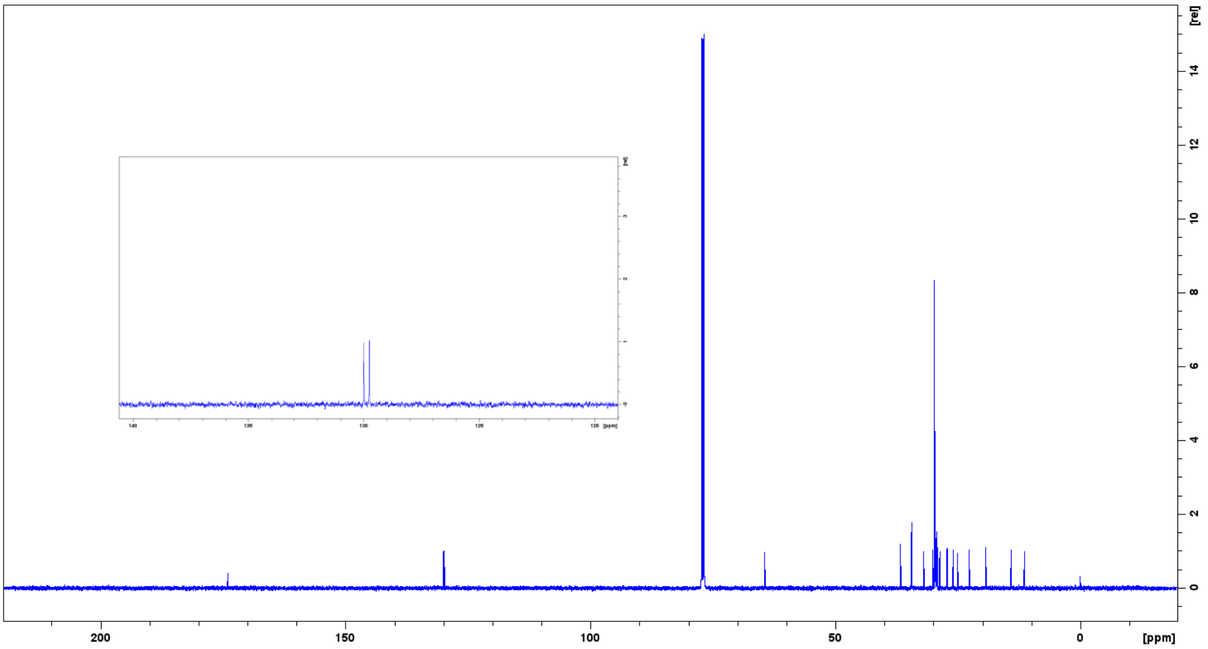

Figure S22. ^{13}C NMR (125.68 MHz) of *anteiso*-$C_{25:0}$/$C_{18:1}$ WE (**13**) at 25 °C.

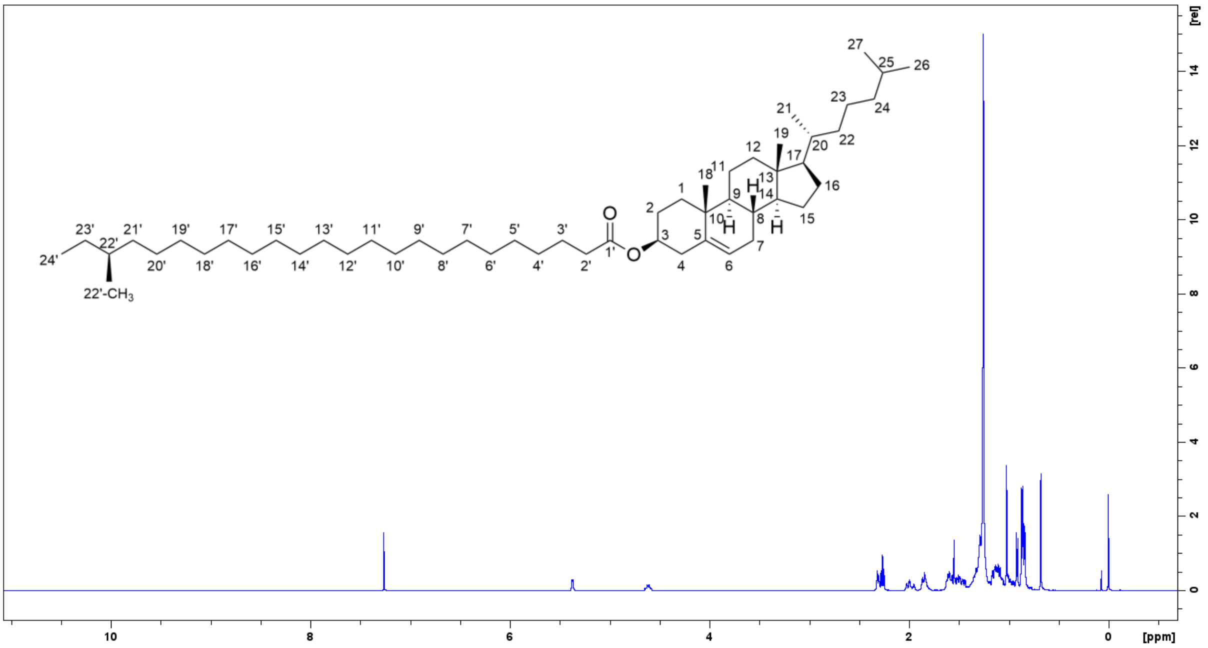

Figure S23. ^{1}H NMR (499.82 MHz) of *anteiso*-$C_{25:0}$ CE (**14**) at 25 °C.

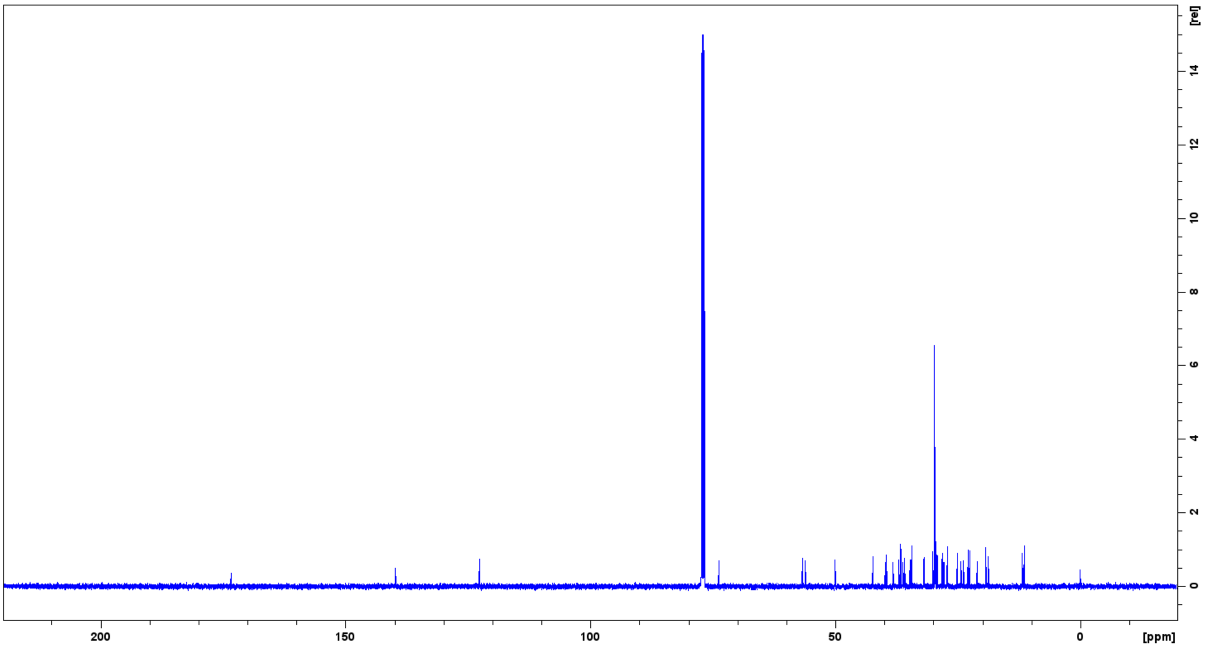

Figure S24. ^{13}C NMR (125.68 MHz) of *anteiso*-$C_{25:0}$ CE (**14**) at 25 °C.

2. Additional Biophysical Data

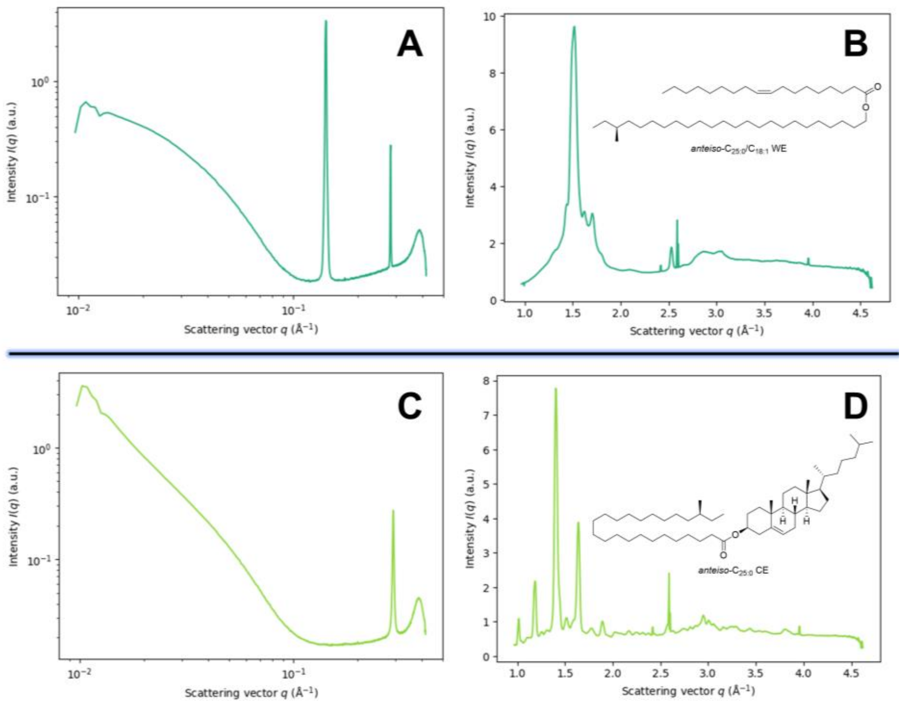

Figure S25. SAXS (**A** = *anteiso*-$C_{25:0}$/$C_{18:1}$WE; **13**, and **C** = *anteiso*-$C_{25:0}$ CE; **14**) and WAXS (**B** = *anteiso*-$C_{25:0}$/$C_{18:1}$ WE; **13**, and **D** = *anteiso*-$C_{25:0}$ CE; **14**) data from the ForMAX beamline at the MAX IV Synchrotron was used to confirm the assigned peak indexes and the associated long spacing values.

Table S1. Overview of melting points and long spacing values of straight-chain, *iso*-branched, *anteiso*-branched WEs and CEs, and bulk human meibum.

Species	Melting Point	Long Spacing
n-$C_{26:0}$/$C_{18:1}$ WE[ϕ]	48.0 °C	99.3 ± 0.4 Å
iso-$C_{26:0}$/$C_{18:1}$ WE[ϕ]	36.8 °C	85.3 ± 0.2 Å
anteiso-$C_{25:0}$/$C_{18:1}$ WE (**13**)	27.7 °C	42.7 ± 1.0 Å
n-$C_{26:0}$ CE[ϕ]	90.9 °C	131.5 ± 0.3 Å
iso-$C_{26:0}$ CE[ϕ]	66.8 °C	104.6 ± 1.0 Å
anteiso-$C_{25:0}$ CE (**14**)	82.5 °C	22.0 ± 1.0 Å
Bulk human meibum (phase A, prevalence 8/10)[ξ]	40–42 °C	110.6 ± 2.3 Å
Bulk human meibum (phase B, prevalence 10/10)[ξ]	33–35 °C	48.8 ± 0.53 Å
Bulk human meibum (phase C, prevalence 5/10)[ξ]	32–35 °C	43.4 ± 0.43 Å

[ϕ] Reference values of straight-chain and *iso*-branched WEs and CEs taken from the publication by Viitaja et.al. in *Colloids Surf. B: Biointerfaces* **2022**, *214*, 112429.

[ξ] Reference values of bulk human meibum taken from the publication by Leiske et. al. in *Biophys.J.* **2012**, *102* (2), 369–376.
