# Supplementary figures and images for: Another Brick in the Wall of Tear Film Insights Added Through the Total Synthesis and Biophysical Profiling of anteiso-Branched Wax and Cholesteryl Esters
Source: J Nat Prod. 2024 Mar 28;87(4):954–65. doi: 10.1021/acs.jnatprod.3c01247 (PMC11389978; doi:10.1021/acs.jnatprod.3c01247)

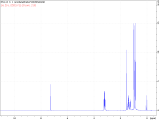

Supplement: Supplementary file 2 — np3c01247_si_002.zip [file np3c01247_si_002.zip › 01/1/pdata/1/thumb.png]

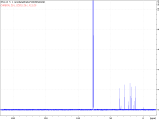

Supplement: Supplementary file 2 — np3c01247_si_002.zip [file np3c01247_si_002.zip › 01/2/pdata/1/thumb.png]

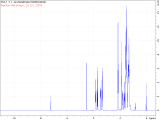

Supplement: Supplementary file 2 — np3c01247_si_002.zip [file np3c01247_si_002.zip › 03/1/pdata/1/thumb.png]

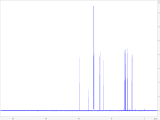

Supplement: Supplementary file 2 — np3c01247_si_002.zip [file np3c01247_si_002.zip › 03/2/pdata/1/thumb.png]

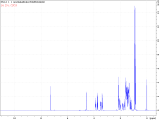

Supplement: Supplementary file 2 — np3c01247_si_002.zip [file np3c01247_si_002.zip › 04/1/pdata/1/thumb.png]

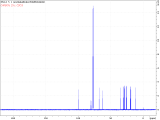

Supplement: Supplementary file 2 — np3c01247_si_002.zip [file np3c01247_si_002.zip › 04/2/pdata/1/thumb.png]

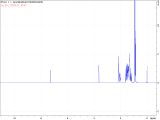

Supplement: Supplementary file 2 — np3c01247_si_002.zip [file np3c01247_si_002.zip › 05/1/pdata/1/thumb.png]

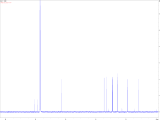

Supplement: Supplementary file 2 — np3c01247_si_002.zip [file np3c01247_si_002.zip › 05/2/pdata/1/thumb.png]
